# Machine learning predictive models and risk factors for lymph node metastasis in non-small cell lung cancer

**DOI:** 10.1186/s12890-024-03345-7

**Published:** 2024-10-22

**Authors:** Bo Wu, Yihui Zhu, Zhuozheng Hu, Jiajun Wu, Weijun Zhou, Maoyan Si, Xiying Cao, Zhicheng Wu, Wenxiong Zhang

**Affiliations:** 1 Department of Thoracic Surgery, The Second Affiliated Hospital, Jiangxi Medical College, Nanchang University, Nanchang, China; 2 Department of Cardiac Surgery, The First Affiliated Hospital, Jiangxi Medical College, Nanchang University, Nanchang, China; 3 School of Data Science, Chinese University of Hong Kong (Shenzhen), Shenzhen, China; 4 Department of Thoracic Surgery, The First Affiliated Hospital of Gannan Medical University, 23 Qingnian Road, Zhanggong District, Ganzhou, Jiangxi 341000 China

**Keywords:** Non-small cell lung cancer, Lymph node metastasis, Risk factors, Generalized linear model, Machine learning, SEER

## Abstract

**Background:**

The prognosis of non-small cell lung cancer (NSCLC) is substantially affected by lymph node metastasis (LNM), but there are no noninvasive, inexpensive methods of relatively high accuracy available to predict LNM in NSCLC patients.

**Methods:**

Clinical data on NSCLC patients were obtained from the Surveillance, Epidemiology, and End Results (SEER) database. Risk factors for LNM were recognized LASSO and multivariate logistic regression. Six predictive models were constructed with machine learning based on risk factors. The area under the receiver operating characteristic curve (AUC) was used to assess the performance of the model. Subgroup analysis with different T-stages was performed on an optimal model. A webpage LNM risk calculator for optimal model was built using the Shinyapps.io platform.

**Results:**

We enrolled 64,012 NSCLC patients, of whom 26,611 (41.57%) had LNM. Using multivariate logistic regression, we finally identified 10 independent risk factors for LNM: age, sex, race, histology, primary site, grade, T stage, M stage, tumor size, and bone metastases. GLM is the optimal model among all six machine learning models in both the training and validation cohorts. Subgroup analyses revealed that GLM has good predictability for populations with different T staging. A webpage LNM risk calculator based on GLM was posted on the shinyapps.io platform (https://wubopredict.shinyapps.io/dynnomapp/).

**Conclusion:**

The predictive model based on GLM can be used to precisely predict the probability of LNM in NSCLC patients, which was proven effective in all subgroup analyses according to T staging.

**Supplementary Information:**

The online version contains supplementary material available at 10.1186/s12890-024-03345-7.

## Introduction

Globally, lung cancer is one of the top malignant tumors in terms of incidence and mortality. Statistically, in 2022, lung cancer will bring an enormous cancer burden to the United States [1]. The pathological types of lung cancer are divided into two main types: non-small cell lung cancer (NSCLC) and small cell lung cancer, with NSCLC accounting for the major portion (85%) [2]. The five-year survival rate for NSCLC is only 26.4%, mainly because most patients are found to have metastasis [3]. Lymph nodes are one of the common sites of metastasis, and lymph node metastasis (LNM) seriously affects the survival time of NSCLC patients. TNM staging clearly reflects this perspective [4]. Unfortunately, although there are many clinical ways to evaluate patients for LNM, each of these methods has some drawbacks. Therefore, developing a new way to predict LNM in NSCLC patients with relative accuracy is warranted.

Early detection and aggressive treatment of LNM in NSCLC can greatly improve the prognosis of patients, and currently, PET-CT is considered to be the best method to assess the status of lymph nodes. However, the misdiagnosis rate and false-negative rate are still concerns. In addition, the high cost and high dose of radiation of PET-CT are unacceptable to many patients [5]. Although ultrasound-guided biopsy and mediastinoscopy for the evaluation of lymph nodes are superior to imaging tests, the disadvantage is that these are invasive tests and carry some risks [6]. These methods are also mentioned in the guidelines. Due to the condition of the clinical equipment and the skill level of the doctors, in practice, most clinicians do not perform this evaluation [7]. Having noninvasive and inexpensive ways to arrive at early LNM diagnoses could help clinicians make the best treatment decisions, whether surgical or combination therapy [8]. Hence, there is an urgent need to establish new models to predict whether LNM occurs in NSCLC patients.

Machine learning (ML) prediction models are relatively mature predictive techniques that are widely used in various disciplines and medicine [9]. In the field of clinical medicine, ML has been used many times to predict the prognosis and distant metastasis of breast, prostate, and liver cancers with good results [10–12]. Currently, there is no ML prediction model to predict LNM in NSCLC.

Hence, we developed six novel models with the ML algorithm for predicting LNM in NSCLC patients based on the Surveillance, Epidemiology, and End Results (SEER) database. Then, an optimal prediction model was selected, and subgroup analysis with different T-stages was performed on the optimal model.

## Methods

### Data source and patient selection

In our study, we downloaded the clinical data of patients with a pathologic diagnosis of NSCLC from the SEER via SEER*stat software (version: 8.4.1). Patient data from the SEER database are freely available worldwide. The study was exempted from the requirement to obtain informed consent by the hospital ethics committee.

**Fig. 1:**
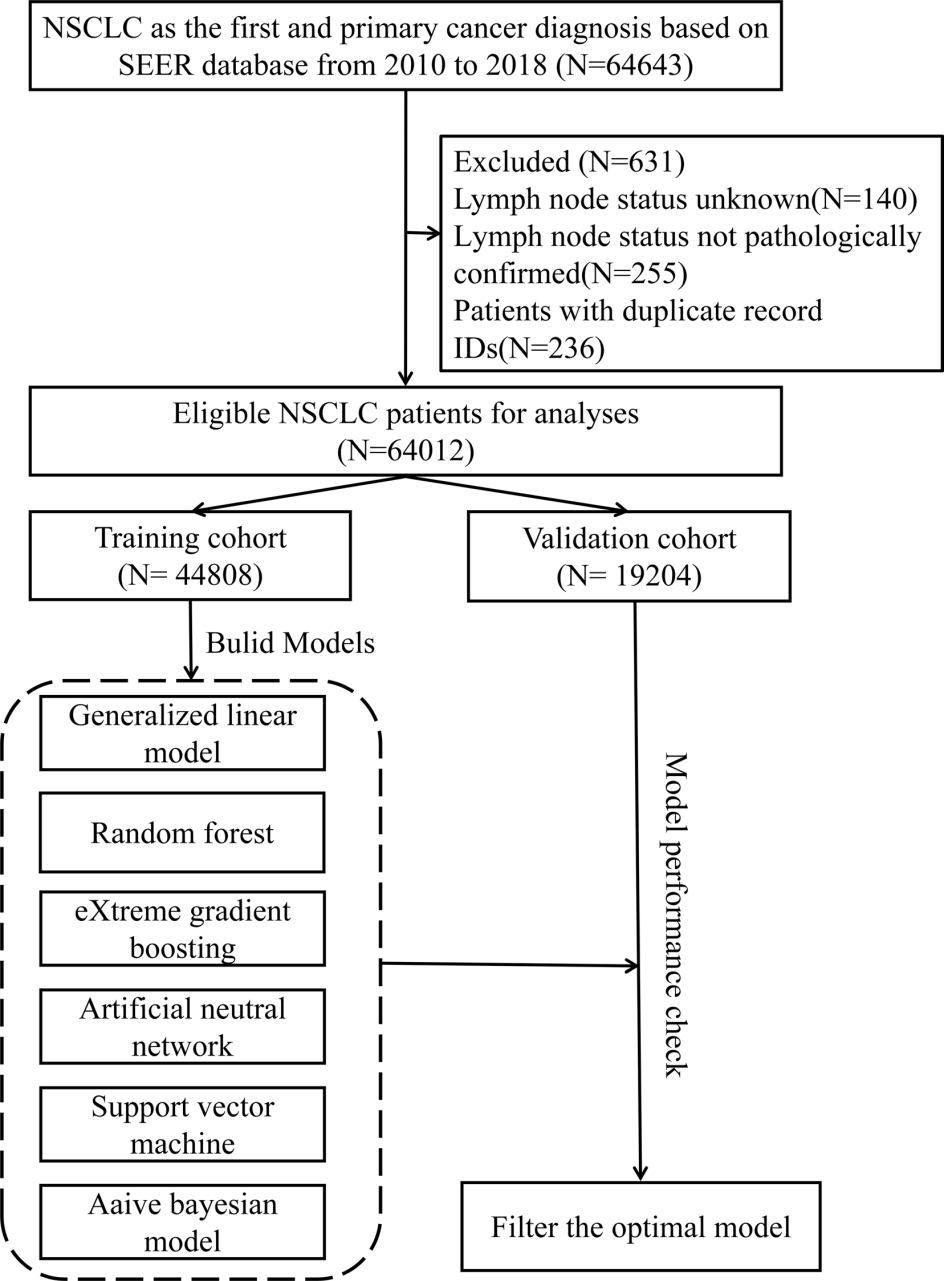
The flow chart of non-small cell lung cancer inclusion population

The inclusion criteria were as follows: (I) pathological type of cancer was NSCLC; (II) the years of diagnosis were from 2010 to 2018; (III) complete demographic and clinical characterization data were available; and (IV) NSCLC was the first primary tumor. The exclusion criteria were as follows: (I) lymph node status not pathologically confirmed; (II) lymph node status unknown; and (III) NSCLC patients with multiple duplicate record IDs. A flowchart for the inclusion of NSCLC patients is displayed in Fig. 1.

## Study variables

Important demographic and clinical characteristics were as follows: age, sex, race, histology, primary site, grade, T stage, M stage, tumor size, bone metastases, brain metastases, liver metastases and lung metastases. Age was classified as “<67 years” or “≥67 years.” Tumor size was classified as “<29 mm” or “≥29 mm.” In this study, the missing value for this variable was treated as a separate category during data analysis [13], as this situation is also likely to arise in clinical practice. The presence of LNM at first diagnosis was the primary outcome of this study.

### Statistical analysis

Kaplan‒Meier curves were utilized to contrast the overall and cancer-specific survival in NSCLC patients with and without LNM. Identification of optimal cutoff values for age and tumor size was accomplished via receiver operating characteristic curve (ROC) analysis [14]. Enrolled patients were randomized into training and validation cohorts at a ratio of 7:3. The Chi-square test was utilized for baseline information for the training and validation cohorts. LASSO regression analysis was used to screen significant variables for LNM in NSCLC patients. Candidate independent risk factors were then identified by multivariate logistic regression analysis. Evaluating the correlation between independent risk factors via correlation analysis. These independent risk factors are used as alternative variables to construct six ML prediction models (generalized linear model (GLM), random forest (RF), extreme gradient boosting (XGB), artificial neutral network (ANN), support vector machine (SVM) and naive Bayesian model (NBM). 10-fold cross-validation in internal validation. Evaluate the importance of variables using the varlmp function. Rank the importance of variables in different ML models. The best model was constructed after excluding variables with a relative importance value of 0% [15]. All of these predictive models utilize the most basic hyperparameters (**Text S1)**.

The predictive ability of these ML models was assessed by means of ROC and the area under the ROC curve (AUC) in the validation cohort. In addition, sensitivity, specificity and accuracy were also indicators for evaluating the ML algorithms. Subgroup analyses were used to validate the performance of the best model in populations with different T stages. The probability density function (PDF) and clinical utility curve (CUC) were utilized to estimate the predictive performance of the optimal models [16]. *P* < 0.05 was deemed statistically significant, and all statistical analyses were performed using the corresponding packages of R software (version 4.0.5). We published the webpage LNM risk calculator via the shinyapps.io platform.

## Results

### Patient characteristics

This study recruited a total of 64,012 patients. There were no significant differences between included (*N* = 64012) and excluded (*N* = 631) patients in terms of demographics and clinical characteristics (Table S1). All variables were not significantly different in the total cohort (*N* = 64012), training cohort (*N* = 44808) and validation cohort (*N* = 19204) (Table 1). In addition, LNM occurred in 26,611 (41.57%) of the included patients, and 37,401 (58.43%) had no LNM. Variables were significantly different between the lymph node-negative and lymph node-positive groups (Table S2).

**Table 1:** Characteristics of NSCLC patients in the training cohort and the validation cohort

Characteristic	Total cohort		Training cohort		Validation cohort	P value
	n = 64,012(100%)		n = 44,808(70%)		n = 19,204(30%)	
**Age**						0.889
<67	30,759 (48.05%)		21,503 (47.99%)		9256 (48.20%)	
≥67	33,253 (51.95%)		23,305 (52.01%)		9948 (51.80%)	
**Sex**						0.717
Female	33,055 (51.64%)		23,091 (51.53%)		9964 (51.89%)	
Male	30,957 (48.36%)		21,717 (48.47%)		9240 (48.11%)	
**Race**						0.996
White	52,392 (81.85%)		36,693 (81.89%)		15,699 (81.75%)	
Black	6544 (10.22%)		4559 (10.17%)		1985 (10.34%)	
Asian	4751 (7.42%)		3332 (7.44%)		1419 (7.39%)	
American Indian	325 (0.51%)		224 (0.50%)		101 (0.53%)	
**Histology**						0.934
LUAD	38,100 (59.52%)		26,628 (59.43%)		11,472 (59.74%)	
SCC	16,967 (26.51%)		11,885 (26.52%)		5082 (26.46%)	
Others	8945 (13.97%)		6295 (14.05%)		2650 (13.8%)	
**Primary site**						0.069
Upper lobe	37,655 (58.82%)		26,340 (58.78%)		11,315 (58.92%)	
Middle lobe	3662 (5.72%)		2554 (5.70%)		1108 (5.77%)	
Lower lobe	20,555 (32.11%)		14,419 (32.18%)		6136 (31.95%)	
Main bronchus	1318 (2.06%)		882 (1.97%)		436 (2.27%)	
Others	822 (1.28%)		613 (1.37%)		209 (1.09%)	
**Grade**						0.989
I	8448 (13.20%)		5904 (13.18%)		2544 (13.25%)	
II	20,672 (32.29%)		14,503 (32.37%)		6169 (32.12%)	
III	17,371 (27.14%)		12,168 (27.16%)		5203 (27.09%)	
IV	776 (1.21%)		555 (1.24%)		221 (1.15%)	
Unknown	16,745 (26.16%)		11,678 (26.06%)		5067 (26.39%)	
**T stage**						0.963
T1	25,025 (39.09%)		17,535 (39.13%)		7490 (39%)	
T2	22,604 (35.31%)		15,765 (35.18%)		6839 (35.61%)	
T3	9933 (15.52%)		6991 (15.60%)		2942 (15.32%)	
T4	6450 (10.08%)		4517 (10.08%)		1933 (10.07%)	
**M stage**						0.668
M0	54,267 (84.78%)		38,024 (84.86%)		16,243 (84.58%)	
M1	9745 (15.22%)		6784 (15.14%)		2961 (15.42%)	
**Tumor size (mm)**						0.999
<29	31,479 (49.18%)		22,037 (49.18%)		9442 (49.17%)	
≥29	32,533 (50.82%)		22,771 (50.82%)		9762 (50.83%)	
**Bone metastases**						0.108
No	60,540 (94.58%)		42,439 (94.71%)		18,101 (94.26%)	
Yes	3280 (5.12%)		2247 (5.01%)		1033 (5.38%)	
Unknown	192 (0.30%)		122 (0.27%)		70 (0.36%)	
**Brain metastases**						0.954
No	60,828 (95.03%)		42,589 (95.05%)		18,239 (94.98%)	
Yes	2975 (4.65%)		2078 (4.64%)		897 (4.67%)	
Unknown	209 (0.33%)		141 (0.31%)		68 (0.35%)	
**Liver metastases**						0.452
No	62,645 (97.86%)		43,863 (97.89%)		18,782 (97.80%)	
Yes	1147 (1.79%)		804 (1.79%)		343 (1.79%)	
Unknown	220 (0.34%)		141 (0.31%)		79 (0.41%)	
**Lung metastases**						0.891
No	61,097 (95.45%)		42,773 (95.46%)		18,324 (95.42%)	
Yes	2667 (4.17%)		1869 (4.17%)		798 (4.16%)	
Unknown	248 (0.39%)		166 (0.37%)		82 (0.43%)	
**Lymph node metastases**						0.89
No	37,401 (58.43%)		26,208 (58.49%)		11,193 (58.28%)	
Yes	26,611 (41.57%)		18,600 (41.51%)		8011 (41.72%)	

**Abbreviations**: LUAD: Lung adenocarcinoma; NSCLC: Non-small cell lung cancer; SCC: Squamous cell carcinoma

### Survival analysis and cutoff value selection

**Fig. 2:**
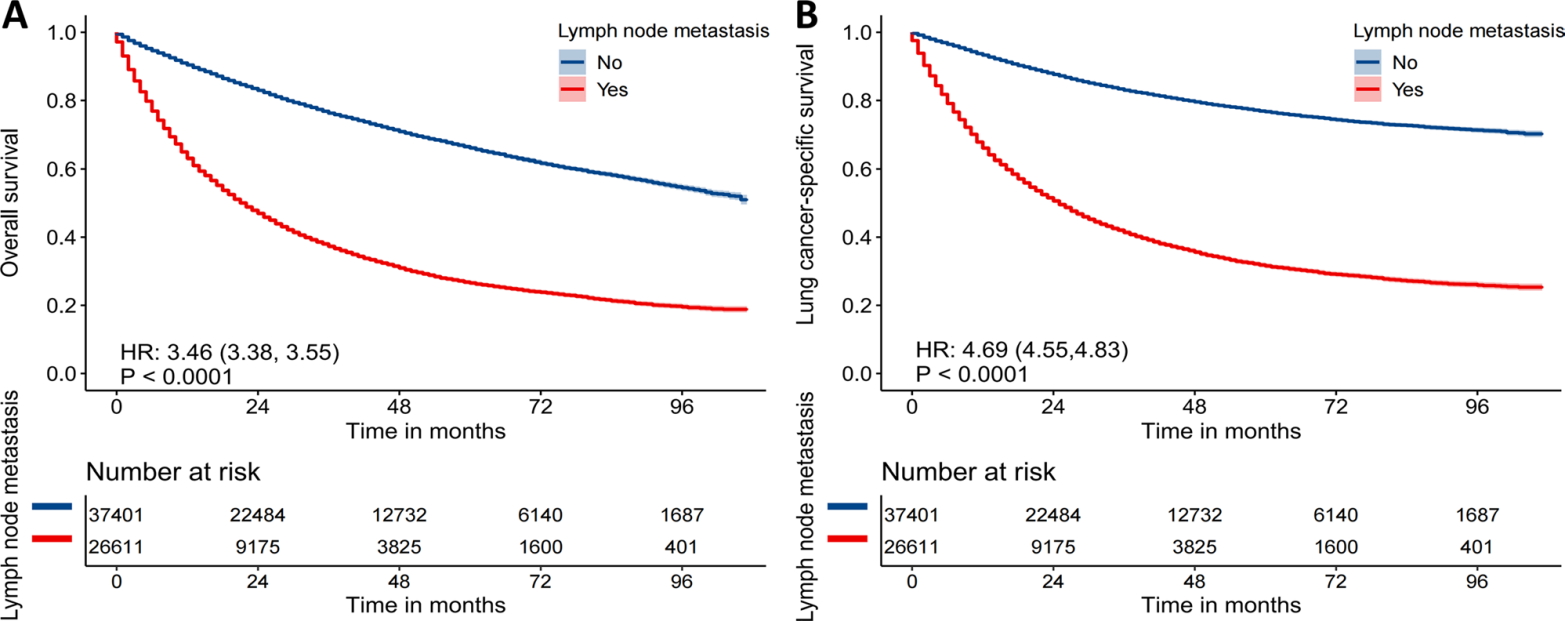
Kaplan‒Meier curves of (**A**) overall survival and (**B**) cancer specific survival for NSCLC patients with and without LNM

Kaplan‒Meier curve results showed that LNM patients had significant associations with overall survival (HR = 3.46, 95%CI (3.38–3.55), *P* < 0.0001 Fig. 2A) and lung cancer-specific survival (HR = 4.69, 95%CI (4.55–4.83), *P* < 0.0001 Fig. 2B). The ROC curves showed cutoff values of 66.5 years and 28.5 mm for age and tumor size, respectively (Figure S1).

## Risk factors for LNM in patients with NSCLC

**Fig. 3:**
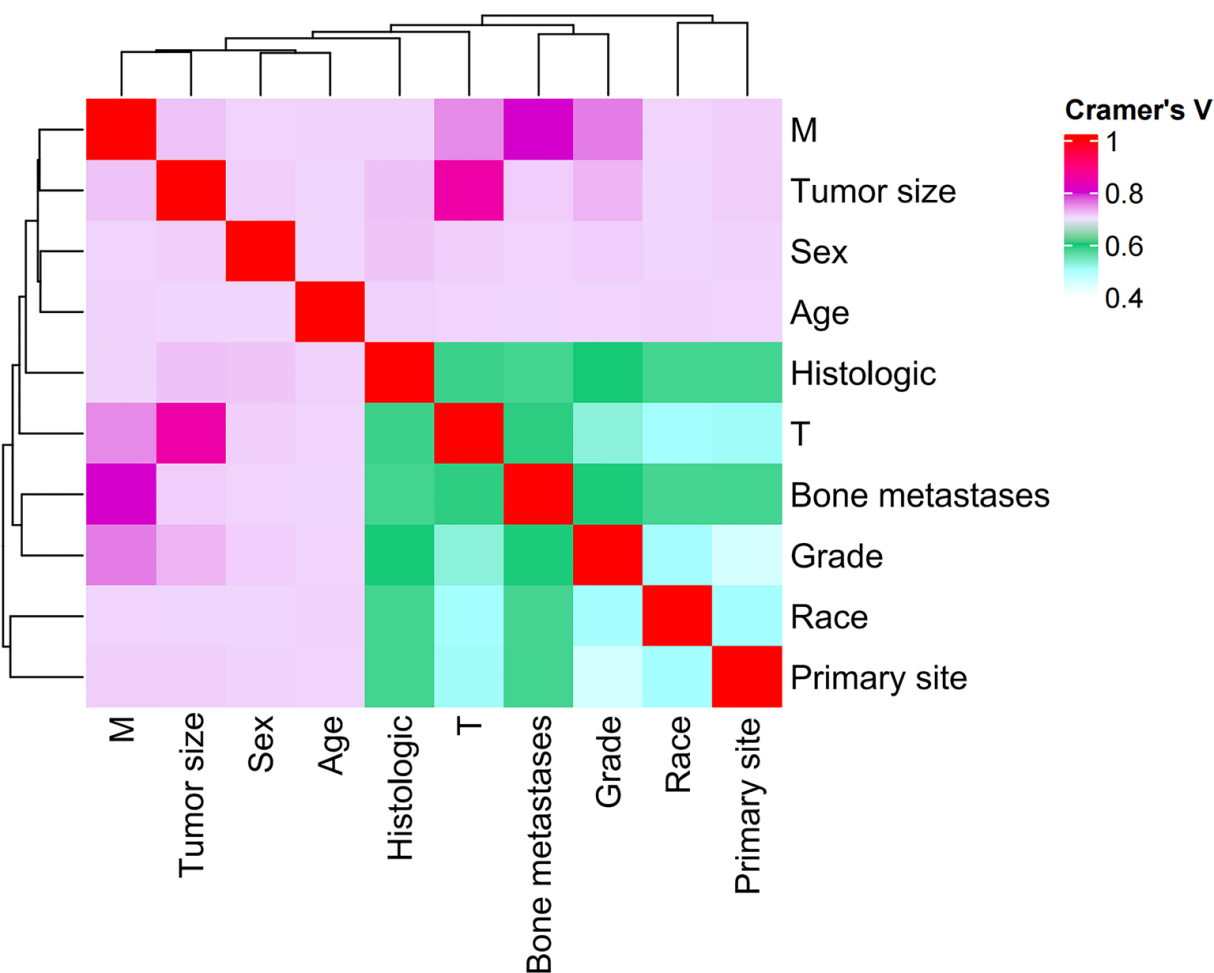
The heatmap of correlation of risk factors

LASSO regression analysis excluded liver metastases and lung metastases, and the 11 unexcluded variables were used for further multivariate logistic regression (Figure S2). The analysis showed 10 variables, including age, sex, race, histology, primary site, grade, T stage, M stage, tumor size and bone metastases, as independent risk factors for LNM in NSCLC (Table 2). The correlation heatmap derived from Cramer’s V correlation analysis indicated that there was no significant relationship between the 10 independent risk factors (Fig. 3).

**Table 2:** Multivariate logistic regression analysis of risk factors for LNM in NSCLC patients

Characteristic	OR (95%CI)	*P* value
**Age**		
<67	Ref	Ref
≥67	0.80(0.76,0.84)	< 0.001
**Sex**		
Female	Ref	Ref
Male	1.14(1.09,1.19)	< 0.001
**Race**		
White	Ref	Ref
Black	1.11(1.03,1.19)	0.009
Asian or Pacific Islander	1.12(1.03,1.23)	0.008
American Indian	1.39(1.02,1.88)	0.036
**Histology**		
LUAD	Ref	Ref
SCC	0.84(0.80,0.89)	< 0.001
Others	0.69(0.64,0.74)	< 0.001
**Primary site**		
Upper lobe	Ref	Ref
Middle lobe	0.95(0.86,1.05)	0.328
Lower lobe	1.08(1.03,1.13)	0.003
Main bronchus	2.16(1.79,2.61)	< 0.001
Others	1.12(0.94,1.35)	0.207
**Grade**		
I	Ref	Ref
II	2.64(2.40,2.89)	< 0.001
III	3.92(3.57,4.31)	< 0.001
IV	3.39(2.75,4.16)	< 0.001
Unknown	9.93(9.01,10.93)	< 0.001
**T stage**		
T1	Ref	Ref
T2	1.51(1.41,1.61)	< 0.001
T3	1.99(1.84,2.15)	< 0.001
T4	3.27(2.96,3.61)	< 0.001
**M stage**		
M0	Ref	Ref
M1	4.74(4.26,5.27)	< 0.001
**Tumor size (mm)**		
<29	Ref	Ref
≥29	1.86(1.75,1.98)	< 0.001
**Bone metastases**		
No	Ref	Ref
Yes	2.51(2.05,3.07)	< 0.001
Unknown	0.95(0.45,2.01)	0.899
**Brain metastases**		
No	Ref	Ref
Yes	1.00(0.85,1.18)	0.981
Unknown	1.69(0.80,3.58)	0.169

**Abbreviations**: CI: Confidence interval; HR: Hazard ratio; LNM: Lymph node metastasis; LUAD: Lung adenocarcinoma; NSCLC: Non-small cell lung cancer; SCC: Squamous cell carcinoma

## Importance of risk factors in the model

**Fig. 4:**
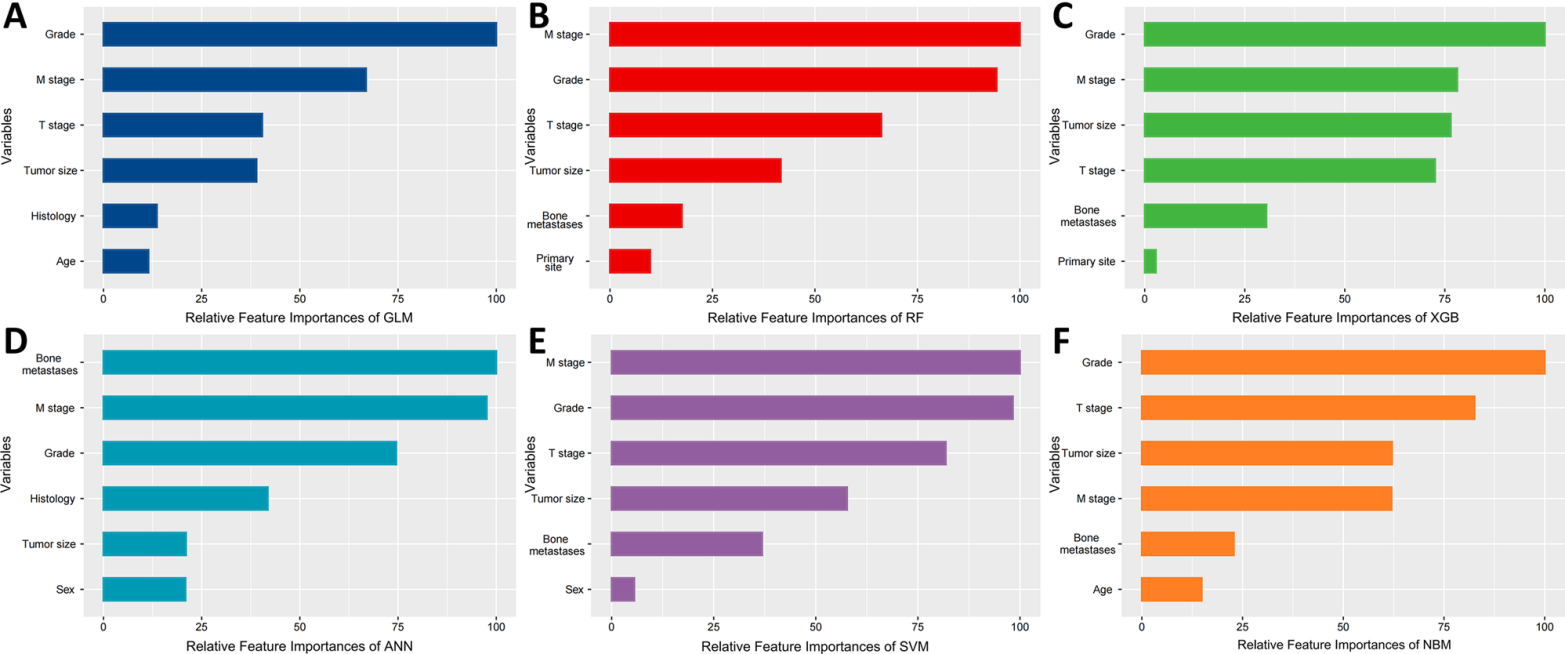
Variable importance of different models. (**A**) Generalized linear model (GLM), (**B**) Random Forest (RF), (**C**) eXtreme gradient boosting (XGB), (**D**) Artificial neutral network (ANN), (**E**) Support vector machine (SVM) and (**F**) Naive Bayesian model (NBM)

ML prediction models were constructed using the 10 independent risk factors mentioned above and the importance of these independent risk factors was calculated (Table S3). The order of contribution of the first six important risk factors in the machine prediction model is depicted in Fig. 4, and the number of risk factors included in each predictive model is summarized in Table S4. Grade was ranked first in importance in 3 out of 6 models, which suggests that grade has a significant effect on LNM in NSCLC patients. Grade, M, T, and tumor size ranked in the top four in importance in most models.

### Building ML predictive models and validation

**Fig. 5:**
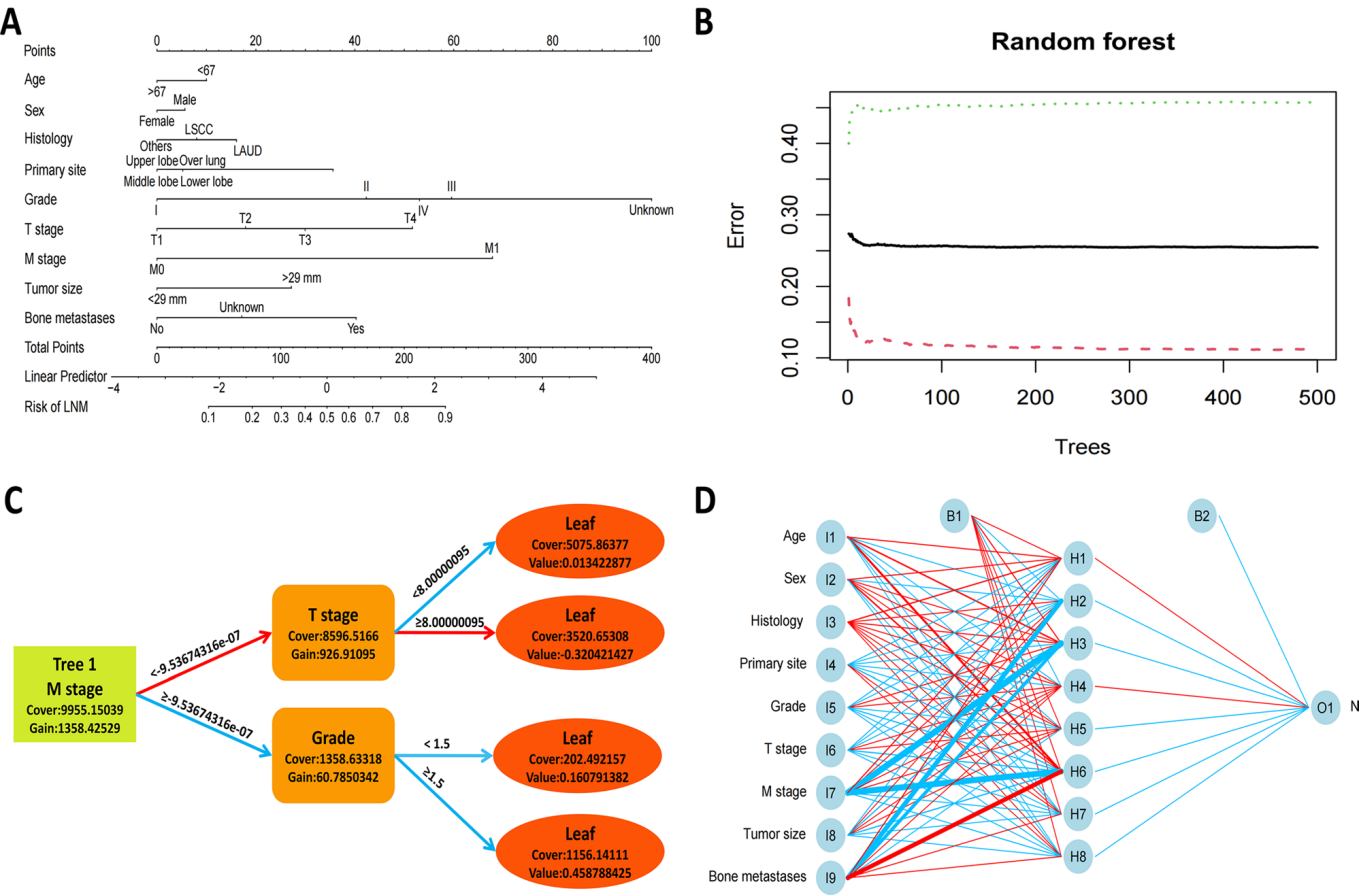
Results of different models. (**A**) A nomogram for predicting the risk of bone metastasis. (**B**) Relationship of dynamic changes between the prediction error and the number of decision trees. (**C**) The gradient boosting decision trees for extreme gradient boosting (XGB) models. (**D**) The selected structure for the artificial neural network (ANN)

**Fig. 6:**
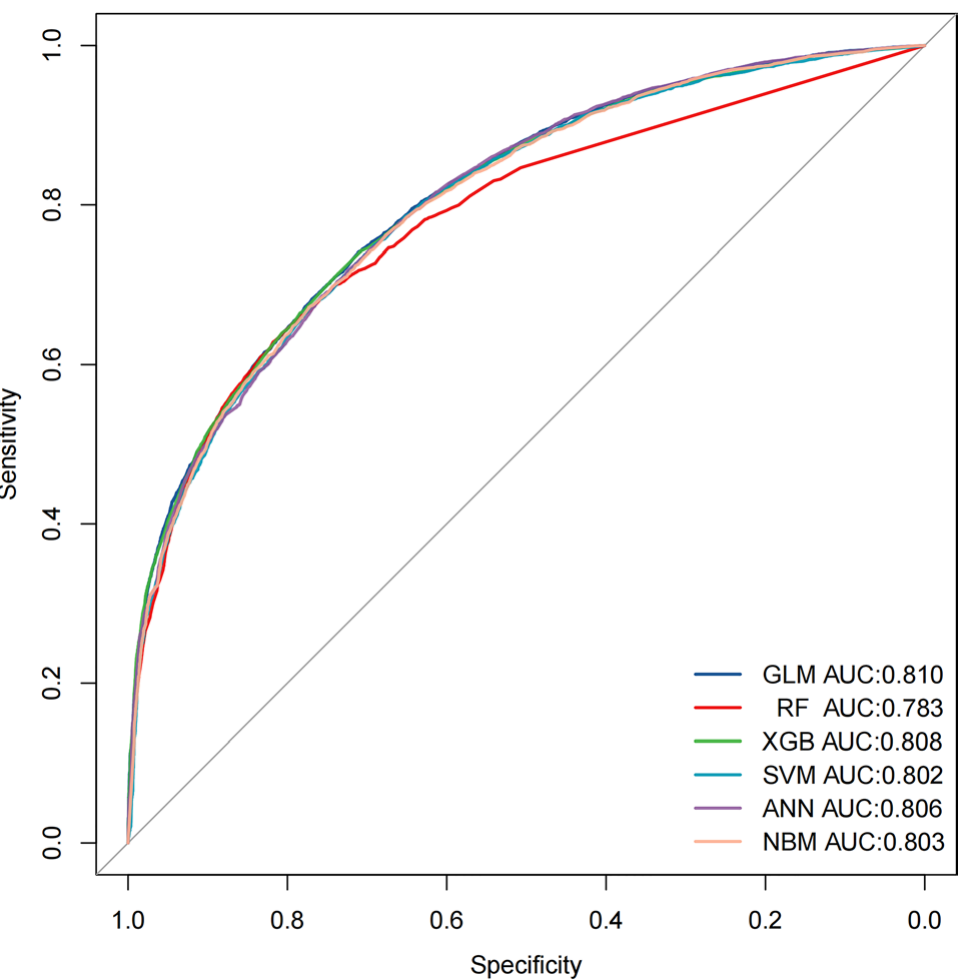
The receiver operating characteristics curves of different machine learning models in the validation cohort

Six ML prediction models were constructed using the above important risk factors. The nomogram model of the GLM is shown in Fig. 5A. Random forests with 464 decision trees were better at distinguishing NSCLC patients with and without LNM (Fig. 5B). Significant effects of the three important variables, including M stage, grade, and T stage, on LNM in NSCLC patients under the decision tree model (Fig. 5C). The ANN model has three layers of neural network, in which the input nodes are 9, the hidden nodes are 8 and the output nodes are 1 (Fig. 5D). Figure S3 shows the relationship between the top six risk factors and LNM. The performance of the six machine prediction models in the training and validation cohorts is depicted in Figure S4,6 and Table S5-6. The ROC curves showed that the GLM has the best predictive ability in the training (AUC = 0.811, 95%CI (0.807 − 0.815)) and validation (AUC = 0.810, 95%CI (0.803 − 0.816)) cohorts. And the GLM model had better sensitivity, specificity and accuracy relative to the remaining five models Fig. 6.

### The best threshold probability of the GLM

PDF and CUC of GLM are decisive for accurate predictions, and there is partial overlap between the two curves in the PDF, with no LNM patients predominantly distributed in the fraction of 0-40.7% risk of metastasis and LNM patients predominantly distributed in the fraction of 40.7–100.0% risk (Figure S5A). The CUC denotes the percentage probability of true positivity for LNM and no LNM at an arbitrary threshold (Figure S5A). In the clinic, an accurate diagnosis of LNM is as important as an accurate diagnosis of no LNM. According to our analysis, 40.7% is used as the threshold probability for clinicians to make a judgment. The probability that patients with no LNM and patients with LNM could be accurately predicted was 71.20% and 73.20%, respectively (Figure S5B).

### Subgroup analyses and web prediction application construction

**Fig. 7:**
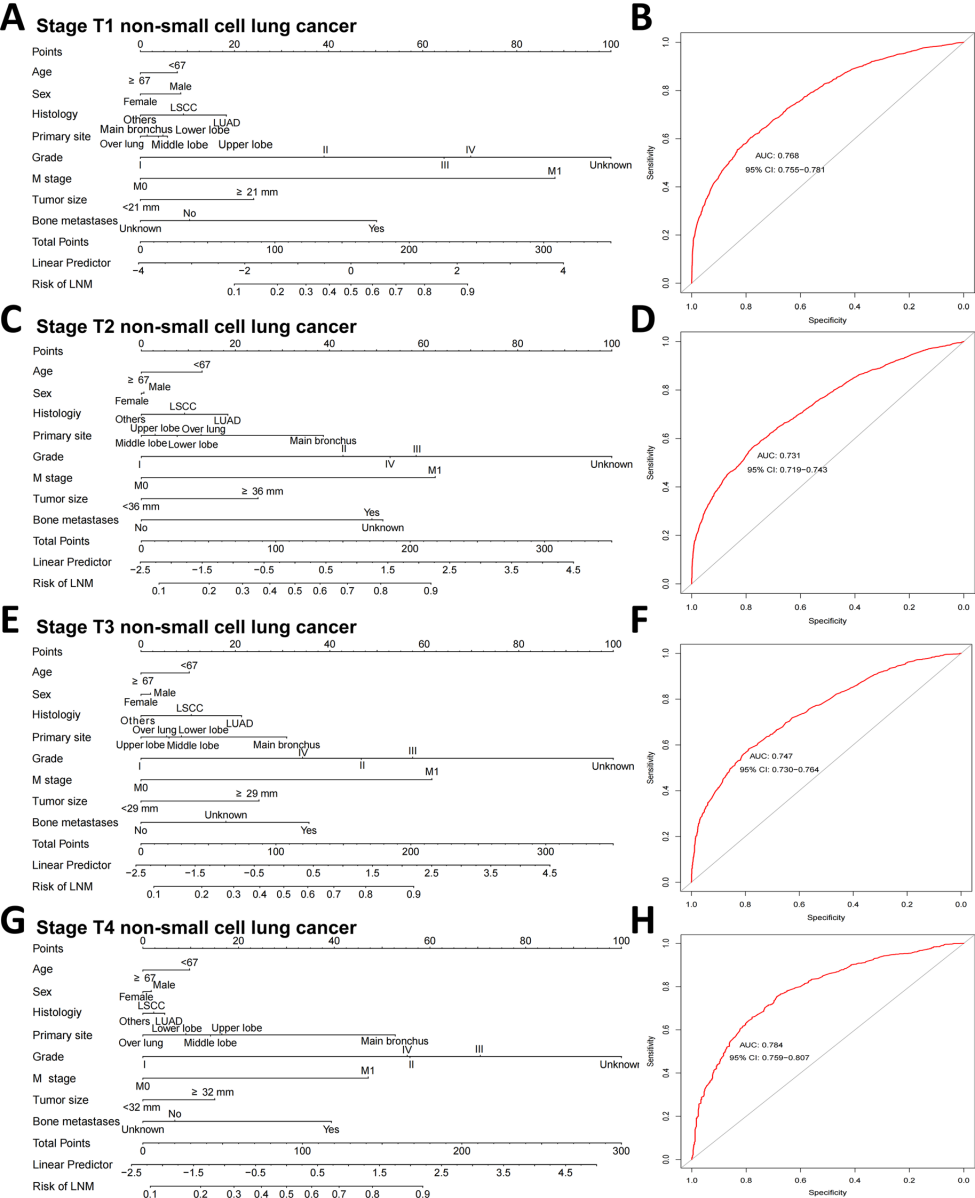
The nomogram for predicting the risk of LNM and ROC curves in stage (**A**, **B**) T1, (**C**, **D**) T2, (**E**, **F**) T3, and (**G**, **H**) T4

**Fig. 8:**
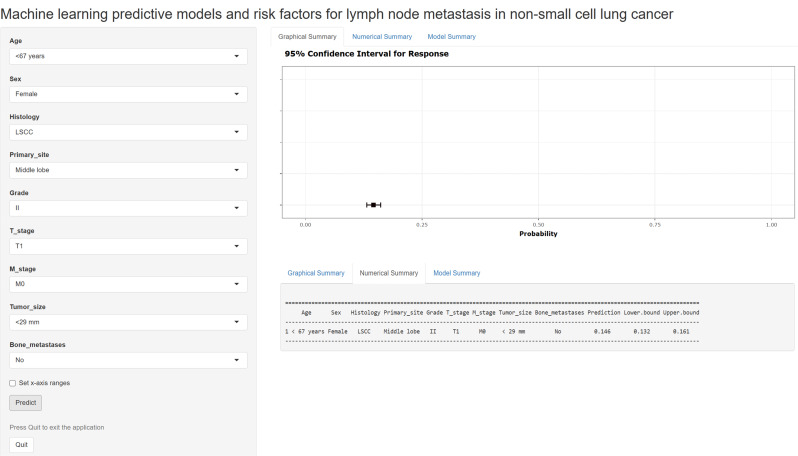
The webpage LNM risk calculator for patients with NSCLC

Subgroup analyses revealed that the AUCs for this model in the T1, T2, T3 and T4 populations were 0.768, 0.731, 0.747 and 0.784, respectively. In addition, the sensitivity, specificity and accuracy suggest that the GLM model has some predictive ability (Fig. 7, Table S7). A webpage LNM risk calculator was developed with the GLM prediction model at the shinyapps.io platform (https://wubopredict.shinyapps.io/dynnomapp/). Clinicians can enter patient risk factors to obtain the probability of a patient’s LNM via a mobile device. The calculator is simple, easy to use and very helpful for doctors to determine the best treatment strategy. We calculated that the LNM probability for a patient (age: 53 years, sex: female, histology: lung squamous cell carcinoma, primary site: middle lobe, grade: II, T stage: T1, M stage: M0, tumor size: < 29 mm, bone metastases: no) was 14.6% (95% CI: 13.2–16.1%) (Fig. 8).

## Discussion

NSCLC poses an enormous cancer burden globally due to its high incidence and mortality rates [1]. The five-year survival rate of NSCLC is as low as 26.4%, mainly because most patients already have metastases at the time of diagnosis [3]. NSCLC patients with or without LNM are significantly affected by prognosis [17]. Unfortunately, there is no noninvasive, inexpensive, and relatively accurate method to diagnose whether a patient has LNM. ML is an important component of artificial intelligence that recognizes variables and complex relationships between them [18, 19]. ML prediction of lymph node metastasis has been well established in kidney, breast, and colorectal cancers [20]. Therefore, developing ML prediction models to predict LNM in NSCLC is necessary. In this study, we identified independent risk factors for LNM in patients with NSCLC and developed six different ML prediction models. We ML models with the best predictive ability and then performed subgroup analysis in different T-stage populations. The clinical effect thresholds for the GLM determined by PDF and CUC enabled accurate identification of NSCLC patients with or without LNM.

In our study, six different ML prediction models were constructed for predicting LNM in NSCLC based on independent risk factors. All models had good predictive ability, and five of the models had AUC values > 0.8. The GLM (AUC: 0.810) had the best predictive performance for LNM. Our GLM has better predictive ability relative to the nomogram of Tian et al. (AUC: 0.810 vs. 0.721) [21]. Subgroup analyses revealed that the GLM has good predictability for populations with different T staging. Although the gold standard for diagnosing LNM in NSCLC is pathologic biopsy, this is mainly used in patients with high clinical suspicion of LNM and is a high-risk invasive test [22]. The advantage of the GLM is that it is applicable to all NSCLC patients and noninvasive. In addition, the advantages of the GLM over PET-CT are low radiation and low cost of the test [23]. Finally, there are many current predictive models that are limited to paper form and have not been developed for practical applications [24, 25], and the GLM webpage LNM risk calculator we built with the shinyapps.io platform quickly helps physicians calculate the probability of a patient’s LNM. Hence, our GLM is a highly accurate, noninvasive, inexpensive and convenient method to predict LNM in NSCLC patients. This is beneficial to help clinicians develop rational treatment strategies and improve patient prognosis.

Multivariate logistic regression was used to finalize the 10 risk factors for LNM, and significant differences were most pronounced for grade, M, T and tumor size. In our study, the risk of grade II, III and IV LNM was 2.64, 3.92 and 3.39 times higher than that of grade I, which is consistent with previous reports that the grading of NSCLC is closely associated with LNM [26]. Patients with worse T-staging and M-staging were more likely to develop lymph node metastases. This is consistent with previous studies [27, 28]. Early studies have found that tumor size greater than 3 cm was a risk factor for LNM, which is similar to our finding of a significantly increased risk of LNM in tumor size ≥ 29 mm [29]. NSCLC patients aged < 67 years are more likely to have LNM. Chen B et al. also found a greater risk of LNM in younger NSCLC patients [30]. We found that males may be more likely to have LNM than females. However, differences in LNM risk by sex remain controversial, with different studies coming to different conclusions [31]. Lung adenocarcinoma carries a greater risk rate of LNM than squamous cell carcinoma [32]. Regarding racial factors, the risk of LNM is highest among Blacks. The reason for this may be the result of possible socioeconomic and genetic factors, among others [33, 34]. NSCLC of the main bronchus is more prone to LNM, which may be related to the anatomy and needs to be further explored. The above independent risk factors reflected their contribution in six ML prediction models, which is consistent with our logistic regression results. Overall, these risk factors significantly affect LNM in NSCLC patients and are useful in assessing LNM.

In this study, we developed a new model based on the GLM algorithm to predict LNM in NSCLC patients with SEER database data. The strengths of the study include the following: First, this is a study of a large population with high accuracy of findings. Second, this was the first ML-based predictive model for predicting LNM in NSCLC patients. Third, our GLM is a good noninvasive, inexpensive way to predict LNM in NSCLC patients with relative accuracy. Fourth, clinicians can quickly and easily calculate the probability of a patient’s lymph node metastasis via the webpage LNM risk calculator. Certainly, some limitations exist. First, this was a retrospective study based on the SEER database. Second, the SEER database lacks some important LNM risk factors, such as smoking status, economic base, and mutation type, the inclusion of which could improve the performance of our predictive models. Third, this study included populations mainly from the United States, and we cannot yet suggest that the model is appropriate for other countries and races around the world. Fourth, this study only did internal validation and lacked effective external validation. In the future, we will conduct a prospective study while incorporating additional beneficial risk variables to validate and enhance the accuracy of our model.

## Conclusion

In conclusion, we found that the GLM had the best performance among six different ML algorithms; its ability to precisely predict the probability of LNM in NSCLC patients was proven by all subgroup analyses according to T staging. Meanwhile, we determined that age, sex, race, histology, primary site, grade, T stage, M stage, tumor size, and bone metastases were independent risk factors for LNM in NSCLC patients. Due to identifiable shortcomings of our study, we need to validate it by conducting a large prospective study in the future.

## Electronic supplementary material

Below is the link to the electronic supplementary material.

Supplementary Material 1

Supplementary Material 2

Supplementary Material 3

Supplementary Material 4

Supplementary Material 5

Supplementary Material 6

Supplementary Material 7

Supplementary Material 8

Supplementary Material 9

Supplementary Material 10

Supplementary Material 11

Supplementary Material 12

Supplementary Material 13

## Data Availability

No datasets were generated or analysed during the current study.

## Abbreviations

ANN: Artificial neutral network
AUC: Area under curve
CI: Confidence interval
CUCs: Clinical utility curves
GLM: Generalized linear model
HR: Hazard ratio
LNM: Lymph node metastasis
NBM: Naive Bayesian model
NSCLC: Non-small cell lung cancer
PDFs: Probability density functions
RF: Random Forest
ROC: Receiver operating characteristic curve
SEER: the Surveillance Epidemiology and End Results database
SVM: Support vector machine
XGB: eXtreme gradient boosting
