# Supplementary material for: Machine learning predictive models and risk factors for lymph node metastasis in non-small cell lung cancer
Source: BMC Pulm Med. 2024 Oct 22;24:526. doi: 10.1186/s12890-024-03345-7 (PMC11515794; doi:10.1186/s12890-024-03345-7)
Supplement: Supplementary file 6 — Supplementary Material 6 [file 12890_2024_3345_MOESM6_ESM.docx]

Table S1 Characteristics of included and excluded patients.

| **Characteristic** | **Inclusion cohort (N=64012)** | **Exclusion cohort (N=631)** | **P value** |
| --- | --- | --- | --- |
| **Age** |  |  | 0.198 |
| <67 | 30760 (48.05%) | 287 (45.48%) |  |
| ≥67 | 33252 (51.95%) | 344 (54.52%) |  |
| **Sex** |  |  | 0.239 |
| Female | 33055 (51.64%) | 311 (49.29%) |  |
| Male | 30957 (48.36%) | 320 (50.71%) |  |
| **Race** |  |  | 0.828 |
| White | 52392 (81.85%) | 516 (81.77%) |  |
| Black | 6544 (10.22%) | 60 (9.51%) |  |
| Asian | 4751 (7.42%) | 52 (8.24%) |  |
| American Indian | 325 (0.51%) | 3 (0.48%) |  |
| **Histology** |  |  | 0.285 |
| LUAD | 38100 (59.52%) | 356 (56.42%) |  |
| SCC | 16967 (26.51%) | 179 (28.37%) |  |
| Others | 8945 (13.97%) | 96 (15.21%) |  |
| **Primary site** |  |  | 0.386 |
| Upper lobe | 37655 (58.82%) | 367 (58.16%) |  |
| Middle lobe | 3662 (5.72%) | 42 (6.66%) |  |
| Lower lobe | 20555 (32.11%) | 199 (31.54%) |  |
| Main bronchus | 1318 (2.06%) | 18 (2.85%) |  |
| Others | 822 (1.28%) | 5 (0.79%) |  |
| **Grade** |  |  | 0.726 |
| I | 8448 (13.2%) | 84 (13.31%) |  |
| II | 20672 (32.29%) | 192 (30.43%) |  |
| III | 17371 (27.14%) | 186 (29.48%) |  |
| IV | 776 (1.21%) | 7 (1.11%) |  |
| Unknown | 16745 (26.16%) | 162 (25.67%) |  |
| **T stage** |  |  | 0.956 |
| T1 | 25025 (39.09%) | 251 (39.78%) |  |
| T2 | 22604 (35.31%) | 223 (35.34%) |  |
| T3 | 9933 (15.52%) | 93 (14.74%) |  |
| T4 | 6450 (10.08%) | 64 (10.14%) |  |
| **M stage** |  |  | 0.436 |
| M0 | 54267 (84.78%) | 542 (85.9%) |  |
| M1 | 9745 (15.22%) | 89 (14.1%) |  |
| **Tumor size (mm)** |  |  | 0.861 |
| <29 | 31529 (49.25%) | 313 (49.6%) |  |
| ≥29 | 32483 (50.75%) | 318 (50.4%) |  |
| **Bone metastases** |  |  | 0.315 |
| No | 60540 (94.58%) | 595 (94.29%) |  |
| Yes | 3280 (5.12%) | 36 (5.71%) |  |
| Unknown | 192 (0.3%) | 0 (0%) |  |
| **Brain metastases** |  |  | 0.355 |
| No | 60828 (95.03%) | 602 (95.4%) |  |
| Yes | 2975 (4.65%) | 29 (4.6%) |  |
| Unknown | 209 (0.33%) | 0 (0%) |  |
| **Liver metastases** |  |  | 0.129 |
| No | 62645 (97.86%) | 615 (97.46%) |  |
| Yes | 1147 (1.79%) | 16 (2.54%) |  |
| Unknown | 220 (0.34%) | 0 (0%) |  |
| **Lung metastases** |  |  | 0.450 |
| No | 61097 (95.45%) | 608 (96.35%) |  |
| Yes | 2667 (4.17%) | 22 (3.49%) |  |
| Unknown | 248 (0.39%) | 1 (0.16%) |  |

**Abbreviations:** LUAD: Lung adenocarcinoma; NSCLC: Non-small cell lung cancer; SCC: Squamous cell carcinoma.
