# Supplementary material for: Machine learning predictive models and risk factors for lymph node metastasis in non-small cell lung cancer
Source: BMC Pulm Med. 2024 Oct 22;24:526. doi: 10.1186/s12890-024-03345-7 (PMC11515794; doi:10.1186/s12890-024-03345-7)
Supplement: Supplementary file 7 — Supplementary Material 7 [file 12890_2024_3345_MOESM7_ESM.docx]

Table S2 Baseline table of NSCLC patients with and without LNM.

| **Characteristic** | **Overall** |  | **No LNM** |  | **LNM** | **P value** |
| --- | --- | --- | --- | --- | --- | --- |
|  | **n=64012(100%)** |  | **n=37401(58.43%)** |  | **n=26611(41.57%)** |  |
| **Age** |  |  |  |  |  | <0.001 |
| <67 | 30759 (48.05%) |  | 16787 (44.88%) |  | 13972 (52.5%) |  |
| ≥67 | 33253 (51.95%) |  | 20614 (55.12%) |  | 12639 (47.5%) |  |
| **Sex** |  |  |  |  |  | <0.001 |
| Female | 33055 (51.64%) |  | 20533 (54.9%) |  | 12522 (47.06%) |  |
| Male | 30957 (48.36%) |  | 16868 (45.1%) |  | 14089 (52.94%) |  |
| **Race** |  |  |  |  |  | <0.001 |
| White | 52392 (81.85%) |  | 31050 (83.02%) |  | 21342 (80.2%) |  |
| Black | 6544 (10.22%) |  | 3356 (8.97%) |  | 3188 (11.98%) |  |
| Asian | 4751 (7.42%) |  | 2821 (7.54%) |  | 1930 (7.25%) |  |
| American Indian | 325 (0.51%) |  | 174 (0.47%) |  | 151 (0.57%) |  |
| **Histology** |  |  |  |  |  | <0.001 |
| LUAD | 38100 (59.52%) |  | 22021 (58.88%) |  | 16079 (60.42%) |  |
| SCC | 16967 (26.51%) |  | 9323 (24.93%) |  | 7644 (28.72%) |  |
| Others | 8945 (13.97%) |  | 6057 (16.19%) |  | 2888 (10.85%) |  |
| **Primary site** |  |  |  |  |  | <0.001 |
| Upper lobe | 37655 (58.82%) |  | 22022 (58.88%) |  | 15633 (58.75%) |  |
| Middle lobe | 3662 (5.72%) |  | 2287 (6.11%) |  | 1375 (5.17%) |  |
| Lower lobe | 20555 (32.11%) |  | 12349 (33.02%) |  | 8206 (30.84%) |  |
| Main bronchus | 1318 (2.06%) |  | 326 (0.87%) |  | 992 (3.73%) |  |
| Others | 822 (1.28%) |  | 417 (1.11%) |  | 405 (1.52%) |  |
| **Grade** |  |  |  |  |  | <0.001 |
| I | 8448 (13.20%) |  | 7480 (20.00%) |  | 968 (3.64%) |  |
| II | 20672 (32.29%) |  | 14919 (39.89%) |  | 5753 (21.62%) |  |
| III | 17371 (27.14%) |  | 9813 (26.24%) |  | 7558 (28.40%) |  |
| IV | 776 (1.21%) |  | 468 (1.25%) |  | 308 (1.16%) |  |
| Unknown | 16745 (26.16%) |  | 4721 (12.62%) |  | 12024 (45.18%) |  |
| **T stage** |  |  |  |  |  | <0.001 |
| T1 | 25025 (39.09%) |  | 19040 (50.91%) |  | 5985 (22.49%) |  |
| T2 | 22604 (35.31%) |  | 12685 (33.92%) |  | 9919 (37.27%) |  |
| T3 | 9933 (15.52%) |  | 4294 (11.48%) |  | 5639 (21.19%) |  |
| T4 | 6450 (10.08%) |  | 1382 (3.70%) |  | 5068 (19.04%) |  |
| **M stage** |  |  |  |  |  | <0.001 |
| M0 | 54267 (84.78%) |  | 36167 (96.70%) |  | 18100 (68.02%) |  |
| M1 | 9745 (15.22%) |  | 1234 (3.30%) |  | 8511 (31.98%) |  |
| **Tumor size (mm)** |  |  |  |  |  | <0.001 |
| <29 | 31479 (49.18%) |  | 22939 (61.33%) |  | 8540 (32.09%) |  |
| ≥29 | 32533 (50.82%) |  | 14462 (38.67%) |  | 18071 (67.91%) |  |
| **Bone metastases** |  |  |  |  |  | <0.001 |
| No | 60540 (94.58%) |  | 37148 (99.32%) |  | 23392 (87.9%) |  |
| Yes | 3280 (5.12%) |  | 195 (0.52%) |  | 3085 (11.59%) |  |
| Unknown | 192 (0.30%) |  | 58 (0.16%) |  | 134 (0.50%) |  |
| **Brain metastases** |  |  |  |  |  | <0.001 |
| No | 60828 (95.03%) |  | 36963 (98.83%) |  | 23865 (89.68%) |  |
| Yes | 2975 (4.65%) |  | 381 (1.02%) |  | 2594 (9.75%) |  |
| Unknown | 209 (0.33%) |  | 57 (0.15%) |  | 152 (0.57%) |  |
| **Liver metastases** |  |  |  |  |  | <0.001 |
| No | 62645 (97.86%) |  | 37271 (99.65%) |  | 25374 (95.35%) |  |
| Yes | 1147 (1.79%) |  | 72 (0.19%) |  | 1075 (4.04%) |  |
| Unknown | 220 (0.34%) |  | 58 (0.16%) |  | 162 (0.61%) |  |
| **Lung metastases** |  |  |  |  |  | <0.001 |
| No | 61097 (95.45%) |  | 37050 (99.06%) |  | 24047 (90.36%) |  |
| Yes | 2667 (4.17%) |  | 284 (0.76%) |  | 2383 (8.95%) |  |
| Unknown | 248 (0.39%) |  | 67 (0.18%) |  | 181 (0.68%) |  |
| **Survival months** | 23 (9,49) |  | 32 (13,59) |  | 14 (5,32) | <0.001 |
| **Vital status recodes** |  |  |  |  |  | <0.001 |
| Dead | 37944 (59.28%) |  | 27416 (73.30%) |  | 10528 (39.56%) |  |
| Alive | 26068 (40.72%) |  | 9985 (26.7%) |  | 16083 (60.44%) |  |
| **Cause-specific death classification** |  |  |  |  |  | <0.001 |
| Dead | 43433 (67.85%) |  | 31043 (83.00%) |  | 12390 (46.56%) |  |
| Alive | 20579 (32.15%) |  | 6358 (17.00%) |  | 14221 (53.44%) |  |

**Abbreviations:** LNM: Lymph node metastasis; LUAD: Lung adenocarcinoma; NSCLC: Non-small cell lung cancer; SCC: Squamous cell carcinoma.
