# Supplementary material for: Machine learning predictive models and risk factors for lymph node metastasis in non-small cell lung cancer
Source: BMC Pulm Med. 2024 Oct 22;24:526. doi: 10.1186/s12890-024-03345-7 (PMC11515794; doi:10.1186/s12890-024-03345-7)
Supplement: Supplementary file 8 — Supplementary Material 8 [file 12890_2024_3345_MOESM8_ESM.docx]

Table S3 Ranking the importance of variables across six machine learning algorithm models.

| **GLM** | |  | **RF** | |  | **XGB** | |  | **ANN** | |  | **SVM** | |  | **NBM** | |
| --- | --- | --- | --- | --- | --- | --- | --- | --- | --- | --- | --- | --- | --- | --- | --- | --- |
| **Variables** | **Relative importance** |  | **Variables** | **Relative importance** |  | **Variables** | **Relative importance** |  | **Variables** | **Relative importance** |  | **Variables** | **Relative importance** |  | **Variables** | **Relative importance** |
| M | 100.00 |  | M | 100.00 |  | Grade | 100.00 |  | Bone metastases | 100.00 |  | M | 100.00 |  | Grade | 100.00 |
| Grade | 94.33 |  | Grade | 94.33 |  | M | 78.12 |  | M | 97.58 |  | Grade | 98.27 |  | T | 82.59 |
| T | 66.20 |  | T | 66.20 |  | Tumor size | 76.49 |  | Grade | 74.54 |  | T | 81.84 |  | Tumor size | 62.09 |
| Tumor size | 41.66 |  | Tumor size | 41.66 |  | T | 72.57 |  | Histologic | 41.84 |  | Tumor size | 57.66 |  | M | 61.98 |
| Bone metastases | 17.52 |  | Bone metastases | 17.52 |  | Bone metastases | 30.40 |  | Tumor size | 21.01 |  | Bone metastases | 36.86 |  | Bone metastases | 22.83 |
| Histologic | 9.79 |  | Histologic | 9.79 |  | Primary site | 2.80 |  | Sex | 20.89 |  | Sex | 5.55 |  | Age | 14.84 |
| Primary site | 9.58 |  | Primary site | 9.58 |  | Sex | 2.79 |  | Age | 20.62 |  | Age | 2.79 |  | Sex | 14.48 |
| Race | 6.33 |  | Race | 6.33 |  | Race | 0.00 |  | Primary site | 10.39 |  | Histologic | 1.68 |  | Histologic | 5.60 |
| Sex | 1.41 |  | Sex | 1.41 |  | Histologic | 0.00 |  | T | 9.08 |  | Primary site | 0.47 |  | Race | 2.59 |
| Age | 0.00 |  | Age | 0.00 |  | Age | 0.00 |  | Race | 0.00 |  | Race | 0.00 |  | Primary site | 0.00 |

**Abbreviations:** ANN: Artificial neutral network; AUC: Area under curve; GLM: Generalized linear model; NBM: Naive Bayesian model; RF: Random Forest; SVM: Support vector machine; XGB: eXtreme gradient boosting.
