# Supplementary material for: Machine learning predictive models and risk factors for lymph node metastasis in non-small cell lung cancer
Source: BMC Pulm Med. 2024 Oct 22;24:526. doi: 10.1186/s12890-024-03345-7 (PMC11515794; doi:10.1186/s12890-024-03345-7)
Supplement: Supplementary file 9 — Supplementary Material 9 [file 12890_2024_3345_MOESM9_ESM.docx]

Table S4 Important variables in the construction of the six machine learning algorithm models.

| **Model** | **Important variables** | **No. of optimal variables** |
| --- | --- | --- |
|  |  |  |
| **GLM** | Grade, M stage, T stage, tumor size, histology, age, sex, bone metastases, primary site | 9 |
|  |  |  |
| **RF** | M stage, grade, T stage, tumor size, bone metastases, histology, primary site, race, sex | 9 |
|  |  |  |
| **XGB** | Grade, M stage, T stage, tumor size,  bone metastases, primary site, sex | 7 |
|  |  |  |
| **ANN** | Bone metastases, M stage, grade, histology,  tumor size, sex, age, primary site, T stage | 9 |
|  |  |  |
| **SVM** | Grade, T stage, tumor size, M stage,  bone metastases, age, sex, histology, race | 9 |
|  |  |  |
| **NBM** | M stage, grade, T stage, tumor size, bone metastases, sex, age, histology, primary site | 9 |
|  |  |  |

**Abbreviations:** ANN: Artificial neutral network; AUC: Area under curve; GLM: Generalized linear model; NBM: Naive Bayesian model; RF: Random Forest; SVM: Support vector machine; XGB: eXtreme gradient boosting.
