# Supplementary material for: Machine learning predictive models and risk factors for lymph node metastasis in non-small cell lung cancer
Source: BMC Pulm Med. 2024 Oct 22;24:526. doi: 10.1186/s12890-024-03345-7 (PMC11515794; doi:10.1186/s12890-024-03345-7)
Supplement: Supplementary file 10 — Supplementary Material 10 [file 12890_2024_3345_MOESM10_ESM.docx]

Table S5 Predictive performance of the six machine learning algorithm models in the training cohort.

| **Model** | **AUC** | | **Sensitivity** | **Specificity** | **Accuracy** |
| --- | --- | --- | --- | --- | --- |
|  | **Mean** | **95% CI** |  |  |  |
| **GLM** | 0.811 | 0.807−0.815 | 0.823 | 0.709 | 0.736 |
|  |  |  |  |  |  |
| **RF** | 0.790 | 0.786−0.793 | 0.735 | 0.735 | 0.735 |
|  |  |  |  |  |  |
| **XGB** | 0.810 | 0.801−0.814 | 0.763 | 0.738 | 0.746 |
|  |  |  |  |  |  |
| **ANN** | 0.803 | 0.800−0.807 | 0.739 | 0.751 | 0.747 |
|  |  |  |  |  |  |
| **SVM** | 0.804 | 0.800−0.808 | 0.874 | 0.669 | 0.701 |
|  |  |  |  |  |  |
| **NBM** | 0.808 | 0.804−0.812 | 0.725 | 0.747 | 0.739 |
|  |  |  |  |  |  |

**Abbreviations:** ANN: Artificial neutral network; AUC: Area under curve; GLM: Generalized linear model; NBM: Naive Bayesian model; RF: Random Forest; SVM: Support vector machine; XGB: eXtreme gradient boosting.
