# Supplementary material for: Machine learning predictive models and risk factors for lymph node metastasis in non-small cell lung cancer
Source: BMC Pulm Med. 2024 Oct 22;24:526. doi: 10.1186/s12890-024-03345-7 (PMC11515794; doi:10.1186/s12890-024-03345-7)
Supplement: Supplementary file 11 — Supplementary Material 11 [file 12890_2024_3345_MOESM11_ESM.docx]

Table S6 Predictive performance of the six machine learning algorithm models in the validation cohort.

| **Model** | **AUC** | | **Sensitivity** | **Specificity** | **Accuracy** |
| --- | --- | --- | --- | --- | --- |
|  | **Mean** | **95% CI** |  |  |  |
| **GLM** | 0.810 | 0.803−0.816 | 0.816 | 0.708 | 0.734 |
|  |  |  |  |  |  |
| **RF** | 0.783 | 0.777−0.789 | 0.726 | 0.730 | 0.729 |
|  |  |  |  |  |  |
| **XGB** | 0.808 | 0.801−0.814 | 0.756 | 0.733 | 0.740 |
|  |  |  |  |  |  |
| **ANN** | 0.802 | 0.796−0.808 | 0.723 | 0.743 | 0.736 |
|  |  |  |  |  |  |
| **SVM** | 0.806 | 0.800−0.812 | 0.866 | 0.667 | 0.699 |
|  |  |  |  |  |  |
| **NBM** | 0.803 | 0.796−0.809 | 0.669 | 0.659 | 0.661 |
|  |  |  |  |  |  |

**Abbreviations:** ANN: Artificial neutral network; AUC: Area under curve; GLM: Generalized linear model; NBM: Naive Bayesian model; RF: Random Forest; SVM: Support vector machine; XGB: eXtreme gradient boosting.
