# Supplementary material for: Machine learning predictive models and risk factors for lymph node metastasis in non-small cell lung cancer
Source: BMC Pulm Med. 2024 Oct 22;24:526. doi: 10.1186/s12890-024-03345-7 (PMC11515794; doi:10.1186/s12890-024-03345-7)
Supplement: Supplementary file 12 — Supplementary Material 12 [file 12890_2024_3345_MOESM12_ESM.docx]

Table S7 Predicted performance of the GLM model for predicting LNM in T1, T2, T3 and T4 populations.

| **Subgroup** | **AUC** | | **Sensitivity** | **Specificity** | **Accuracy** |
| --- | --- | --- | --- | --- | --- |
|  | **Mean** | **95% CI** |  |  |  |
| **Stage T1** | 0.768 | 0.755−0.781 | 0.821 | 0.805 | 0.806 |
|  |  |  |  |  |  |
| **Stage T2** | 0.731 | 0.719−0.743 | 0.780 | 0.654 | 0.679 |
|  |  |  |  |  |  |
| **Stage T3** | 0.747 | 0.730−0.764 | 0.795 | 0.576 | 0.660 |
|  |  |  |  |  |  |
| **Stage T4** | 0.784 | 0.759−0.807 | 0.856 | 0.475 | 0.772 |
|  |  |  |  |  |  |

**Abbreviations:** AUC: Area under curve; GLM: Generalized linear model.
