# Supplementary material for: Machine learning predictive models and risk factors for lymph node metastasis in non-small cell lung cancer
Source: BMC Pulm Med. 2024 Oct 22;24:526. doi: 10.1186/s12890-024-03345-7 (PMC11515794; doi:10.1186/s12890-024-03345-7)
Supplement: Supplementary file 13 — Supplementary Material 13 [file 12890_2024_3345_MOESM13_ESM.docx]

**Text S1** R language code for machine learning models.

#NOM

#路径

library(Hmisc)

library(grid)

library(lattice)

library(Formula)

library(ggplot2)

setwd("E:\\NSCLC\\7 nomgram")

#加载安装包

library("rms")

library(foreign)

library(survival)

#读取数据-改为自己的数据

seer<-read.table("NSCLC.txt",header=T,sep="\t")

dd <- datadist(seer)

options(datadist="dd")

#将数据集分为训练集和测试集,比例为7:3

set.seed(123)

train_sub = sample(nrow(seer),7/10*nrow(seer))

train_data= seer[train_sub,]

test_data= seer[-train_sub,]

#将数据转换成因子格式

train_data$Age<-factor(train_data$Age,labels=c("<67","≥67"))

train_data$Sex<-factor(train_data$Sex,labels=c("Female","Male"))

train_data$Histologic<-factor(train_data$Histologic,labels=c("LADC","LSCC","Others"))

train_data$Race<-factor(train_data$Race,labels=c("White","Black","Asian or Pacific Islander","American Indian/Alaska Native"))

train_data$Site<-factor(train_data$Site,labels=c("Upper lobe","Middle lobe","Lower lobe","Main bronchus","Over lung"))

train_data$Grade<-factor(train_data$Grade,labels=c("I","II","III","IV","Unknown"))

train_data$T<-factor(train_data$T,labels=c("T1","T2","T3","T4"))

train_data$N<-factor(train_data$N,labels=c("0","1"))

train_data$M<-factor(train_data$M,labels=c("M0","M1"))

train_data$Size<-factor(train_data$Size,labels=c("<29","≥29"))

train_data$Bone<-factor(train_data$Bone,labels=c("No","Yes","Unknown"))

m <- glm(N~Age+Sex+Histologic+Site+Grade+T+M+Size+Bone,data=train_data,family = binomial)

library(rms)#建模包

predictions <- predict(m, train_data)

predicted_prob <- predictions # 模型预测的概率（可以是预测概率、分数或类别标签）

actual <- train_data$N

predicted <- ifelse(predicted_prob >= 0.5, 1, 0)

# 计算混淆矩阵

conf_matrix <- table(predicted, actual)

# 计算灵敏度（Sensitivity）

sensitivity <- conf_matrix[2, 2] / sum(conf_matrix[2, ])

# 计算特异度（Specificity）

specificity <- conf_matrix[1, 1] / sum(conf_matrix[1, ])

# 计算准确率（Accuracy）

accuracy <- sum(diag(conf_matrix)) / sum(conf_matrix)

# 打印结果

cat("Sensitivity:", sensitivity, "\n")

cat("Specificity:", specificity, "\n")

cat("Accuracy:", accuracy, "\n")

#test

predictions <- predict(m, test_data)

predicted_prob <- predictions # 模型预测的概率（可以是预测概率、分数或类别标签）

actual <- test_data$N

predicted <- ifelse(predicted_prob >= 0.5, 1, 0)

# 计算混淆矩阵

conf_matrix <- table(predicted, actual)

# 计算灵敏度（Sensitivity）

sensitivity <- conf_matrix[2, 2] / sum(conf_matrix[2, ])

# 计算特异度（Specificity）

specificity <- conf_matrix[1, 1] / sum(conf_matrix[1, ])

# 计算准确率（Accuracy）

accuracy <- sum(diag(conf_matrix)) / sum(conf_matrix)

# 打印结果

cat("Sensitivity:", sensitivity, "\n")

cat("Specificity:", specificity, "\n")

cat("Accuracy:", accuracy, "\n")

nom<-nomogram(m,fun=plogis,lp=T,funlabel="Risk of Death")

#2列线图

pdf("列线图训练组1.pdf",width=10, height=7, onefile=FALSE)

plot(nom,

#1.变量与图形的占比

xfrac=0.23,

#2.变量字体加粗

cex.var=1,

#3.数轴：字体的大小

cex.axis=0.9,

#4.数轴：刻度的长度

tcl=-0.1,

#5.数轴：文字与刻度的距离

lmgp=0.1,

#6.数轴：刻度下的文字，1=连续显示，2=隔一个显示一个

label.every=2,

#7.1个页面有几5个数轴(这个可以压缩行间距)

naxes=16,

#8.垂直线的颜色.

#col.grid=gray(c(0.8,0.95)),

#9.线性预测轴名字

#lplabel="Linear Predictorlp",

#10变量分数名字

points.label="Points",

#11总分名字

total.points.label="Total Points",

force.label=T)#没啥用。TRUE强制标记的每个刻度线都绘制标签，我也没研究明白

dev.off()

#十倍交叉验证+变量重要性排序

library(caret)

formula.init0 <- as.formula("N ~ Age+Sex+Histologic+Site+Grade+T+M+Size+Bone")

control <- trainControl(method="repeatedcv",number=10,repeats=2)

model <- train(formula.init0,data=train_data,method="glm",trControl=control)

importance <- varImp(model,scale=100)

top<-read.table("top.txt",header=T,sep="\t")

pdf("tops6贡献值.pdf", width=8, height=5, onefile=FALSE)

ggplot(top, aes(x=name, y=overall,fill=name)) +

geom_bar(stat = "identity",fill="#00468BE5",color="#00468BE5",size=1, width = 0.5)+coord_flip()+theme_gray(base_size = 15)

dev.off()

#预测模型的概率密度函数和临床效用曲线

library(data.table)

library(ggpubr)

library(pROC)

library(patchwork)

library(eoffice)

N<-factor(test_data$N,labels=c("N0","N1"))

data=data.table(value=m1,group=N)

value=m1

group=N

#绘制ROC曲线，寻找cutoff

rr=roc(data$group,data$value)

plot(rr,thresholds="best",print.thres="best")

#rr$thresholds

best=0.415

#根据分组生成x轴数值所对应的y轴数值列表

pdata=data[,list(x=density(value)$x,y=density(value)$y),by="group"]

#计算颜色所占概率

N0=nrow(subset(data,group=="N0" & value <0.381))/nrow(subset(data,group=="N0"))

N1=nrow(subset(data,group=="N1" & value >0.381))/nrow(subset(data,group=="N1"))

M11=paste0(100*N0,"%")

N11=paste0(100*N1,"%")

#绘制概率密度图

A=ggplot(data) +

stat_density(aes(x=value,color=group),

geom="line",

position="dodge")+

scale_color_manual(values=c("#0099B4E5","red"))+

geom_ribbon(data=subset(pdata,group=="N0" & x<best),

aes(x=x,ymax=y),ymin=0,fill="#0099B4E5", alpha=0.5)+

geom_ribbon(data=subset(pdata,group=="N1" & x>best),

aes(x=x,ymax=y),ymin=0,fill="#ED0000E5", alpha=0.5)+

geom_vline(xintercept = best,linetype=15,size=1)+

theme_test()+

scale_x_continuous(limits = c(NA,max(value)))+

geom_text(data = data.frame(x=c(median(subset(data,group=="N1")$value),

median(subset(data,group=="N0")$value)),

y=c(max(subset(pdata,group=="N1")$y),

max(subset(pdata,group=="N0")$y)),

t=c(N11,M11)),

aes(x=x,y=y,label=t))

A

B=ggplot(data,aes(x=value,color=group))+

stat_ecdf()+

geom_vline(xintercept = best,linetype=15,size=1)+

theme_test()+scale_color_manual(values=c("#0099B4E5","red"))+

scale_x_continuous(limits = c(NA,max(value)))+

geom_hline(yintercept = c(N1,1-N0),linetype=15,size=1)

B

pdf("概率密度函数和临床效用曲线0.pdf")

library(patchwork)

A+B+plot_layout(ncol = 1,guides = "collect")

dev.off()

#RF

library(openxlsx)

#读取数据-改为自己的数据

setwd("E:\\NSCLC\\7 rf")

seer<-read.table("NSCLC.txt",header=T,sep="\t")

#将数据集分为训练集和测试集,比例为7:3

set.seed(123)

train_sub = sample(nrow(seer),7/10*nrow(seer))

train_data1= seer[train_sub,]

test_data1= seer[-train_sub,]

library(randomForest)

#数据预处理

train_data$N = as.factor(train_data$N)

test_data$N = as.factor(test_data$N)

seer_randomforest <- randomForest(N~Site+Sex+Race+Histologic+Grade+T+M+Size+Bone+Lung+Brain,

data = train_data,

ntree =500,

mtry=3,

importance=TRUE ,

proximity=TRUE)

#绘制模型误差与决策树关系图

pdf(file="forest1.pdf")#width=6, height=6

plot(seer_randomforest, main="Random forest", lwd=2)

dev.off()

print(seer_randomforest)#展示随机森林模型

#查第一次看变量的重要性

pdf("查看第一次变量的重要性.pdf")

seer_randomforest$importance

varImpPlot(seer_randomforest, main = "variable importance")

dev.off()

#找出误差最小的点

optionTrees=which.min(seer_randomforest$err.rate[,1])

optionTrees#最好的树个数

rf=randomForest(N~Site+Sex+Histologic+Race+Grade+T+M+Size+Bone, data=train_data, ntree=464)

importance=importance(x=rf)

#查看变量的重要性

pdf("查看变量的重要性.pdf")

rf$importance

varImpPlot(rf, main = "variable importance")

dev.off()

#对测试集进行预测ROC

library(pROC)

pred1RF <- predict(rf, newdata = test_data,type="prob")

pred_3RF<- ifelse(predicted_prob >= 0.5, 1, 0)

pred_3RF=prob=pred1RF [,1]

modelroc_2RF <- roc( test_data$N,pred_3RF)

modelroc_2RF

ci1RF=ci.auc(modelroc_2RF, method="bootstrap")

ciVecRF=as.numeric(ci1RF)

#1训练集ROC曲线

pdf("绘制ROC曲线testRF测试.pdf")

plot(modelroc_2RF ,col="red",

print.auc=TRUE,

main='ROC曲线')

text(0.39, 0.43, paste0("95% CI: ",sprintf("%.03f",ciVecRF[1]),"-",sprintf("%.03f",ciVecRF[3])), col="blue")

dev.off()

#对测试集进行预测ROC

library(pROC)

pred1RF <- predict(rf, newdata = train_data,type="prob")

pred_3RF=prob=pred1RF [,1]

modelroc_2RF <- roc( train_data$N,pred_3RF)

modelroc_2RF

ci1RF=ci.auc(modelroc_2RF, method="bootstrap")

ciVecRF=as.numeric(ci1RF)

#1训练集ROC曲线

pdf("绘制ROC曲线testRF测试.pdf")

plot(modelroc_2RF ,col="red",

print.auc=TRUE,

main=ROC曲线')

text(0.39, 0.43, paste0("95% CI: ",sprintf("%.03f",ciVec[1]),"-",sprintf("%.03f",ciVec[3])), col="blue")

dev.off()

library(caret)

#十倍交叉验证

control <- trainControl(method="repeatedcv",number=10,repeats = 2)

formula.init0 <- as.formula("N ~ .")

model <- train(formula.init0,data=train_data,method="rf",trControl = control)

importance <- caret::varImp(model,scale=100)

#重要性排序

top<-read.table("top.txt",header=T,sep="\t")

pdf("tops6贡献值.pdf", width=8, height=5, onefile=FALSE)

ggplot(top, aes(x=name, y=overall,fill=name)) +

geom_bar(stat = "identity",fill="#ED0000E5",color="#ED0000E5",size=1, width = 0.5)+coord_flip()+theme_gray(base_size = 15)

dev.off()

predictions <- predict(m, train_data)

predicted_prob <- predictions # 模型预测的概率（可以是预测概率、分数或类别标签）

actual <- train_data$N

predicted <- ifelse(predicted_prob >= 0.5, 1, 0)

# 计算混淆矩阵

conf_matrix <- table(predicted, actual)

# 计算灵敏度（Sensitivity）

sensitivity <- conf_matrix[2, 2] / sum(conf_matrix[2, ])

# 计算特异度（Specificity）

specificity <- conf_matrix[1, 1] / sum(conf_matrix[1, ])

# 计算准确率（Accuracy）

accuracy <- sum(diag(conf_matrix)) / sum(conf_matrix)

# 打印结果

cat("Sensitivity:", sensitivity, "\n")

cat("Specificity:", specificity, "\n")

cat("Accuracy:", accuracy, "\n")

#test

predictions <- predict(rf, test_data)

predicted_prob <- predictions # 模型预测的概率（可以是预测概率、分数或类别标签）

actual <- test_data$N

predicted <- ifelse(predicted_prob >= 0.5, 1, 0)

# 计算混淆矩阵

conf_matrix <- table(predicted_prob, actual)

# 计算灵敏度（Sensitivity）

sensitivity <- conf_matrix[2, 2] / sum(conf_matrix[2, ])

# 计算特异度（Specificity）

specificity <- conf_matrix[1, 1] / sum(conf_matrix[1, ])

# 计算准确率（Accuracy）

accuracy <- sum(diag(conf_matrix)) / sum(conf_matrix)

# 打印结果

cat("Sensitivity:", sensitivity, "\n")

cat("Specificity:", specificity, "\n")

cat("Accuracy:", accuracy, "\n")

#XGB

#读取数据-改为自己的数据

setwd("E:\\NSCLC\\9 XGboost 极限梯度提升")

seer<-read.table("NSCLC7.txt",header=T,sep="\t")

#将数据集分为训练集和测试集,比例为7:3

set.seed(123)

train_sub = sample(nrow(seer),7/10*nrow(seer))

train_data = seer[train_sub,]

test_data = seer[-train_sub,]

library(xgboost)

library(Matrix)

####训练集的数据预处理

# 将自变量转化为矩阵

traindata1 <- data.matrix(train_data[,c(1:7)])

# 利用Matrix函数，将sparse参数设置为TRUE，转化为稀疏矩阵

traindata2 <- Matrix(traindata1,sparse=T)

traindata3 <- train_data[,8]

# 将自变量和因变量拼接为list

traindata4 <- list(data=traindata2,label=traindata3)

# 构造模型需要的xgb.DM对象，处理对象为稀疏矩阵

dtrain <- xgb.DMatrix(data = traindata4$data, label = traindata4$label)

####测试集的数据预处理

# 将自变量转化为矩阵

testset1 <- data.matrix(test_data[,c(1:7)])

# 利用Matrix函数，将sparse参数设置为TRUE，转化为稀疏矩阵

testset2 <- Matrix(testset1,sparse=T)

# 将因变量转化为numeric

testset3 <- test_data[,8]

# 将自变量和因变量拼接为list

testset4 <- list(data=testset2,label=testset3)

# 构造模型需要的xgb.DMatrix对象，处理对象为稀疏矩阵

dtest <- xgb.DMatrix(data = testset4$data, label = testset4$label)

#xgboost函数

num_round = 2

xgb <- xgboost(data = dtrain,max_depth=2, eta=0.5, objective='binary:logistic', nround=200, verbose = 1, print_every_n = 200,early_stopping_rounds = 200 )

pred1<-predict(xgb, dtrain)

#预测

library(pROC) #绘制ROC曲线

#训练集预测概率

testpredprobXGB <- predict(xgb, dtrain)

#训练集ROC

obs_p_ran = data.frame(prob=testpredprobXGB,obs=train_data$N)

testrocXGB <- roc(response=train_data$N,#实际类别

predictor =testpredprobXGB) #预测概率

table(obs=train_data$N,testpredprobXGB,dnn = c("真实值","预测值"))

#训练集ROC曲线

modelroc_2XGB <- roc( train_data$N,testpredprobXGB)

modelroc_2XGB

ci1XGB=ci.auc(modelroc_2XGB, method="bootstrap")

ciVecXGB=as.numeric(ci1XGB)

predicted_prob <- testpredprobXGB # 模型预测的概率（可以是预测概率、分数或类别标签）

actual <- train_data$N

predicted <- ifelse(predicted_prob >= 0.5, 1, 0)

# 计算混淆矩阵

conf_matrix <- table(predicted, actual)

# 计算灵敏度（Sensitivity）

sensitivity <- conf_matrix[2, 2] / sum(conf_matrix[2, ])

# 计算特异度（Specificity）

specificity <- conf_matrix[1, 1] / sum(conf_matrix[1, ])

# 计算准确率（Accuracy）

accuracy <- sum(diag(conf_matrix)) / sum(conf_matrix)

# 打印结果

cat("Sensitivity:", sensitivity, "\n")

cat("Specificity:", specificity, "\n")

cat("Accuracy:", accuracy, "\n")

#十倍交叉验证+变量重要性排序

library(caret)

formula.init0 <- as.formula("N ~ Age+Sex+Race+Histologic+Site+Grade+T+M+Size+Bone")

control <- trainControl(method="repeatedcv",number=10,repeats=2)

model <- train(formula.init0,data=train_data,method="rpart",trControl=control)

importance <- varImp(model,scale=100)

top<-read.table("top.txt",header=T,sep="\t")

pdf("top6贡献值.pdf", width=8, height=5, onefile=FALSE)

ggplot(top, aes(x=name, y=overall,fill=name)) +

geom_bar(stat = "identity",fill="#42B540E5",color="#42B540E5",size=1,width = 0.5)+coord_flip()+theme_gray(base_size = 15)

dev.off()

#ANN

#读取数据-改为自己的数据

setwd("E:\\NSCLC\\10.neuralNet") #设置工作目录

seer<-read.table("NSCLC.txt",header=T,sep="\t")

str(seer)

normalize <- function(x) { return((x - min(x)) / (max(x) - min(x)))}

# 规范化数据

seer_norm <- as.data.frame(lapply(seer, normalize))

summary(seer_norm$N)

summary(seer$N)

#将数据集分为训练集和测试集,比例为7:3

set.seed(123)

train_sub = sample(nrow(seer),7/10*nrow(seer))

train_data = seer[train_sub,]

test_data = seer[-train_sub,]

train_data <- datadist(train_data)

options(datadist="train_data")

# 训练数据集

library(neuralnet)

library(NeuralNetTools)

library(ggplot2)

library(ggpol)

library(caret)

concrete_model <- neuralnet(formula =N~Age+Sex+Histologic+Site+Grade+T+M+Size+Bone,

data = train_data,hidden = 8, ## 隐藏层神经元数量

act.fct = "logistic", ## 激活函数

linear.output = FALSE,

algorithm = "rprop+",stepmax=1e6)

predictions <- predict(concrete_model, train_data)

predicted_prob <- predictions # 模型预测的概率（可以是预测概率、分数或类别标签）

actual <- train_data$N

predicted <- ifelse(predicted_prob >= 0.5, 1, 0)

# 计算混淆矩阵

conf_matrix <- table(predicted, actual)

# 计算灵敏度（Sensitivity）

sensitivity <- conf_matrix[2, 2] / sum(conf_matrix[2, ])

# 计算特异度（Specificity）

specificity <- conf_matrix[1, 1] / sum(conf_matrix[1, ])

# 计算准确率（Accuracy）

accuracy <- sum(diag(conf_matrix)) / sum(conf_matrix)

# 打印结果

cat("Sensitivity:", sensitivity, "\n")

cat("Specificity:", specificity, "\n")

cat("Accuracy:", accuracy, "\n")

## 可视化模型中变量的重要性

#garson(concrete_model)

#olden(concrete_model)+ggtitle("Variable importance using connection weights")

#预测为0，1

trainpredprob<-predict(concrete_model, train_data)

#预测

library(pROC) #绘制ROC曲线

obs_p_ran = data.frame(prob=trainpredprob,obs=train_data$N)

trainroc <- roc(response=train_data$N,#实际类别

predictor =trainpredprob) #预测概率

table(obs=train_data$N,trainpredprob,dnn = c("真实值","预测值"))

#训练集ROC曲线

modelroc_2 <- roc( train_data$N,trainpredprob)

modelroc_2

ci1=ci.auc(modelroc_2, method="bootstrap")

ciVec=as.numeric(ci1)

#绘图

pdf("绘制ROC曲线train.pdf")

plot(trainroc,col="#00468BE5",

print.auc=TRUE,

main='模型ROC曲线')

text(0.39, 0.43, paste0("95% CI: ",sprintf("%.03f",ciVec[1]),"-",sprintf("%.03f",ciVec[3])), col="blue")

dev.off()

#预测为0，1

trainpredprobANN<-predict(concrete_model, test_data)

#预测

library(pROC) #绘制ROC曲线

obs_p_ran = data.frame(prob=trainpredprobANN,obs=test_data$N)

trainrocANN <- roc(response=test_data$N,#实际类别

predictor =trainpredprobANN) #预测概率

table(obs=test_data$N,trainpredprobANN,dnn = c("真实值","预测值"))

#训练集ROC曲线

modelroc_2ANN <- roc( test_data$N,trainpredprobANN)

modelroc_2ANN

ci1ANN=ci.auc(modelroc_2ANN, method="bootstrap")

ciVecANN=as.numeric(ci1ANN)

#绘图

pdf("绘制ROC曲线test.pdf")

plot(modelroc_2ANN ,col="#00468BE5",

print.auc=TRUE,

main='模型ROC曲线')

text(0.39, 0.43, paste0("95% CI: ",sprintf("%.03f",ciVecANN[1]),"-",sprintf("%.03f",ciVecANN[3])), col="blue")

dev.off()

library(caret)

#十倍交叉验证

control <- trainControl(method="repeatedcv",number=10,repeats = 2)

formula.init0 <- as.formula("N ~ Age+Sex+Race+Histologic+Site+Grade+T+M+Size+Bone")

model <- train(formula.init0,data=train_data,method="nnet",trControl = control)

importance <- caret::varImp(model,scale=100)

#重要性排序

top<-read.table("top.txt",header=T,sep="\t")

pdf("ANN top6贡献值.pdf", width=8, height=5, onefile=FALSE)

ggplot(top, aes(x=name, y=overall,fill=name)) +

geom_bar(stat = "identity",fill="#0099B4E5",color="#0099B4E5",size=1, width = 0.5)+coord_flip()+theme_gray(base_size = 15)

dev.off()

#SVM

library(ggplot2)

#读取数据-改为自己的数据

setwd("E:\\NSCLC\\svm")

seer<-read.table("NSCLC.txt",header=T,sep="\t")

#将数据集分为训练集和测试集,比例为7:3

set.seed(123)

train_sub = sample(nrow(seer),7/10*nrow(seer))

train_data = seer[train_sub,]

test_data = seer[-train_sub,]

library(pROC) #绘制ROC曲线

library(e1071)

#数据预#svm：由于是分类问题，此处我们选择C-classification

svm<- svm(N~Age+Sex+Race+Histologic+Grade+T+M+Size+Bone,

data = test_data,kernel = "linear",cost = 10, scale =FALSE)

train_data$N = as.factor(train_data$N)

test_data$N = as.factor(test_data$N)

predictions <- predict(svm, train_data)

predicted_prob <- predictions # 模型预测的概率（可以是预测概率、分数或类别标签）

actual <- train_data$N

predicted <- ifelse(predicted_prob >= 0.5, 1, 0)

# 计算混淆矩阵

conf_matrix <- table(predicted, actual)

# 计算灵敏度（Sensitivity）

sensitivity <- conf_matrix[2, 2] / sum(conf_matrix[2, ])

# 计算特异度（Specificity）

specificity <- conf_matrix[1, 1] / sum(conf_matrix[1, ])

# 计算准确率（Accuracy）

accuracy <- sum(diag(conf_matrix)) / sum(conf_matrix)

# 打印结果

cat("Sensitivity:", sensitivity, "\n")

cat("Specificity:", specificity, "\n")

cat("Accuracy:", accuracy, "\n")

#训练集ROC

obs_p_ranSVMtrain = data.frame(prob=trainpredprobSVMtrain,obs=train_data$N)

trainroc <- roc(response=train_data$N,#实际类别

predictor =trainpredprobSVMtrain) #预测概率

table(obs=train_data$N,trainpredprobSVMtrain,dnn = c("真实值","预测值"))

#训练集ROC曲线

modelroc_2SVMtrain <- roc( train_data$N,trainpredprobSVMtrain)

modelroc_2SVMtrain

ci1SVMtrain=ci.auc(modelroc_2SVMtrain, method="bootstrap")

ciVecSVMtrain=as.numeric(ci1SVMtrain)

#训练集ROC曲线

pdf("绘制ROC曲线SVMtrain.pdf")

plot(modelroc_2SVMtrain ,col="#925E9FE5",

print.auc=TRUE,

main='模型ROC曲线')

text(0.39, 0.43, paste0("95% CI: ",sprintf("%.03f",ciVecSVMtrain[1]),"-",sprintf("%.03f",ciVecSVMtrain[3])), col="blue")

dev.off()

#训练集ROC

obs_p_ranSVMtest = data.frame(prob=testpredprobSVMtest,obs=test_data$N)

testrocSVMtest <- roc(response=test_data$N,#实际类别

predictor =testpredprobSVMtest) #预测概率

table(obs=test_data$N,testpredprobSVMtest,dnn = c("真实值","预测值"))

#训练集ROC曲线

modelroc_21SVMtest <- roc( test_data$N,testpredprobSVMtest)

modelroc_21SVMtest

ci1SVMtest=ci.auc(modelroc_21SVMtest, method="bootstrap")

ciVecSVMtest=as.numeric(ci1SVMtest)

#训练集ROC曲线

pdf("绘制ROC曲线SVMtest .pdf")

plot(modelroc_21SVMtest ,col="#925E9FE5",

print.auc=TRUE,

main='模型ROC曲线')

text(0.39, 0.43, paste0("95% CI: ",sprintf("%.03f",ciVecSVMtest[1]),"-",sprintf("%.03f",ciVecSVMtest[3])), col="blue")

dev.off()

#十倍交叉验证+变量重要性排序

library(caret)

formula.init0 <- as.formula("N ~ Age+Sex+Race+Histologic+Site+Grade+T+M+Size+Bone")

control <- trainControl(method="repeatedcv",number=10,repeats=2)

model <- train(formula.init0,data=train_data,method="svmRadial",trControl=control)

importance <- varImp(model,scale=100)

top<-read.table("top.txt",header=T,sep="\t")

pdf("top6贡献值.pdf", width=8, height=5, onefile=FALSE)

ggplot(top, aes(x=name, y=overall,fill=name)) +

geom_bar(stat = "identity",fill="#925E9FE5",color="#925E9FE5",size=1,width = 0.5)+coord_flip()+theme_gray(base_size = 15)

dev.off()

library(ggplot2)

#NBM

#读取数据-改为自己的数据

setwd("E:\\NSCLC\\13 贝叶斯")

seer<-read.table("NSCLC.txt",header=T,sep="\t")

#数据预处理

#将数据集分为训练集和测试集,比例为7:3

set.seed(123)

train_sub = sample(nrow(seer),7/10*nrow(seer))

train_data = seer[train_sub,]

test_data = seer[-train_sub,]

train_data$Age<-factor(train_data$Age)

train_data$Sex<-factor(train_data$Sex)

train_data$HistologicType<-factor(train_data$Histologic)

train_data$Race<-factor(train_data$Race)

train_data$Site<-factor(train_data$Site)

train_data$Grade<-factor(train_data$Grade)

train_data$T<-factor(train_data$T)

train_data$N<-factor(train_data$N)

train_data$M<-factor(train_data$M)

train_data$Size<-factor(train_data$Size)

train_data$Bone<-factor(train_data$Bone)

#2.5 朴素贝叶斯

library(pROC) #绘制ROC曲线

library(klaR)

library(e1071)

library(gmodels)

#贝叶斯模型训练

#train_bay<-naiveBayes(train,train$N,laplace = 1)#laplace拉普拉斯平滑

train_bay<-NaiveBayes(N~Age+Sex+Race+Histologic+Grade+T+M+Size+Bone,data=train_data)

train_bay[1:length(train_bay)]

plot(train_bay)

#train

predictions <- predict(train_bay,train_data)

predicted_prob <- predictions$posterior[,2] # 模型预测的概率（可以是预测概率、分数或类别标签）

actual <- train_data$N

predicted <- ifelse(predicted_prob >= 0.5, 1, 0)

# 计算混淆矩阵

conf_matrix <- table(predicted, actual)

# 计算灵敏度（Sensitivity）

sensitivity <- conf_matrix[2, 2] / sum(conf_matrix[2, ])

# 计算特异度（Specificity）

specificity <- conf_matrix[1, 1] / sum(conf_matrix[1, ])

# 计算准确率（Accuracy）

accuracy <- sum(diag(conf_matrix)) / sum(conf_matrix)

# 打印结果

cat("Sensitivity:", sensitivity, "\n")

cat("Specificity:", specificity, "\n")

cat("Accuracy:", accuracy, "\n")

#test

predictions <- predict(train_bay,test_data)

predicted_prob <- predictions$posterior[,2] # 模型预测的概率（可以是预测概率、分数或类别标签）

actual <- test_data$N

predicted <- ifelse(predicted_prob >= 0.5, 1, 0)

# 计算混淆矩阵

conf_matrix <- table(predicted, actual)

# 计算灵敏度（Sensitivity）

sensitivity <- conf_matrix[2, 2] / sum(conf_matrix[2, ])

# 计算特异度（Specificity）

specificity <- conf_matrix[1, 1] / sum(conf_matrix[1, ])

# 计算准确率（Accuracy）

accuracy <- sum(diag(conf_matrix)) / sum(conf_matrix)

# 打印结果

cat("Sensitivity:", sensitivity, "\n")

cat("Specificity:", specificity, "\n")

cat("Accuracy:", accuracy, "\n")

#2.5.2预测

#预测train

pred1trainNBM<-predict(train_bay,train_data,type="prob")

library( C50 )

library(VIM)

library(mice)

library(mlbench)

predictionstrainNBM=pred1trainNBM$posterior[,2]

#2.5.3 模型评估

pred_2trainNBM <- as.ordered(predictionstrainNBM)

modelroc_2trainNBM <- roc( train_data$N,pred_2trainNBM)

modelroc_2trainNBM

ci1trainNBM=ci.auc(modelroc_2trainNBM, method="bootstrap")

ciVectrainNBM=as.numeric(ci1trainNBM)

#训练集ROC曲线

pdf("绘制ROC曲线trainNBM.pdf")

plot(modelroc_2trainNBM ,col="chocolate1",

print.auc=TRUE,

main='模型ROC曲线')

text(0.39, 0.43, paste0("95% CI: ",sprintf("%.03f",ciVectrainNBM[1]),"-",sprintf("%.03f",ciVectrainNBM[3])), col="blue")

dev.off()

#预测test

pred1testNBM<-predict(train_bay,test_data,type="prob")

library( C50 )

library(VIM)

library(mice)

library(mlbench)

predictionstestNBM=pred1testNBM$posterior[,2]

#2.5.3 模型评估

pred_2testNBM <- as.ordered(predictionstestNBM)

modelroc_2testNBM <- roc( test_data$N,pred_2testNBM)

modelroc_2testNBM

ci1testNBM=ci.auc(modelroc_2testNBM, method="bootstrap")

ciVectestNBM=as.numeric(ci1testNBM)

#训练集ROC曲线

pdf("绘制ROC曲线testNBM.pdf")

plot(modelroc_2 ,col="chocolate1",

print.auc=TRUE,

main='模型ROC曲线')

text(0.39, 0.43, paste0("95% CI: ",sprintf("%.03f",ciVec[1]),"-",sprintf("%.03f",ciVec[3])), col="blue")

dev.off()

pdf("ROC.pdf")

modelroc_2trainNBM <- roc( train_data$N,pred_2trainNBM)

modelroc_2testNBM <- roc( test_data$N,pred_2testNBM)

plot(modelroc_2trainNBM, col="chocolate1",

print.auc=TRUE, lwd=2,

main='模型ROC曲线',print.auc.x=0.5,print.auc.y=0.5)

text(0.1, 0.5, paste0("95% CI: ",sprintf("%.03f",ciVectrainNBM[1]),"-",sprintf("%.03f",ciVectrainNBM[3])), col="blue")

plot.roc(modelroc_2testNBM,add=T,col="red",

print.auc=TRUE,

main='模型ROC曲线',print.auc.x=0.6,print.auc.y=0.6)

text(0.1, 0.6, paste0("95% CI: ",sprintf("%.03f",ciVectestNBM[1]),"-",sprintf("%.03f",ciVectestNBM[3])), col="blue")

legend("bottomright", legend=c("NBM AUC:0.812","modelroc_2testNBM"),col=c("chocolate1","red"),lty=1,lwd=2,bty="n")

dev.off()

#十倍交叉验证+变量重要性排序

library(caret)

formula.init0 <- as.formula("N ~ Age+Sex+Race+Histologic+Site+Grade+T+M+Size+Bone")

control <- trainControl(method="repeatedcv",number=10,repeats=2)

model <- train(formula.init0,data=train_data,method="nb",trControl=control)

importance <- varImp(model,scale=100)

top<-read.table("top.txt",header=T,sep="\t")

pdf("top6贡献值 .pdf", width=8, height=5, onefile=FALSE)

ggplot(top, aes(x=name, y=overall,fill=name)) +

geom_bar(stat = "identity",fill="chocolate1",color="chocolate1",size=1,width = 0.5)+coord_flip()+theme_gray(base_size = 15)

dev.off()
